# Autoimmune Reporting Signals in FAERS Reports Listing COVID-19 Vaccines as Suspect Products: An Active-Comparator Disproportionality Analysis

**DOI:** 10.3390/ijerph23081088

**Published:** 2026-08-21

**Authors:** Assylzhan M. Messova, Makhmutbay Sanbayev, Sagira T. Abdrahmanova, Raikhan Temirkhanova, Nadiar M. Mussin, Amin Tamadon

**Affiliations:** 1 Pediatric Diseases Department, Astana Medical University, Astana 010000, Kazakhstan; messova.a@amu.kz (A.M.M.); abdrakhmanova.s@amu.kz (S.T.A.); 2 Department of Traumatology and Pediatric Surgery, Semey Medical University, Semey 071400, Kazakhstan; 3 Department of General Practice, Semey Medical University, Semey 071400, Kazakhstan; 4 Department of General Surgery, West Kazakhstan Marat Ospanov Medical University, Aktobe 030019, Kazakhstan; nadiar_musin@zkmu.kz; 5 Department of Natural Sciences, West Kazakhstan Marat Ospanov Medical University, Aktobe 030019, Kazakhstan; amintamaddon@yahoo.com

**Keywords:** COVID-19 vaccination, FAERS, pharmacovigilance, vaccine safety, autoimmune adverse events, immune-mediated adverse events, disproportionality analysis, reporting odds ratio, MedDRA preferred term, vaccine platform

## Abstract

**Highlights:**

**Public health relevance—How does this work relate to a public health issue?**

- Continuous post-marketing vaccine safety surveillance is essential for detecting rare, heterogeneous, or subgroup-specific adverse-event reporting patterns that may not be fully characterized before vaccine authorization.
- This study uses FAERS as a complementary pharmacovigilance source to evaluate autoimmune and immune-mediated reporting patterns in FAERS reports listing COVID-19 vaccines as suspect products within an active-comparator vaccine reporting framework.

**Public health significance—Why is this work of significance to public health?**

- By applying strict MedDRA Preferred Term definitions and comparing COVID-19 vaccine reports with traditional non-COVID vaccine reports, this analysis helps improve the specificity of autoimmune signal detection in spontaneous reporting data.
- The findings highlight that broad autoimmune categories may mask or dilute clinically specific Preferred Term-level signals, emphasizing the need for refined case definitions in vaccine pharmacovigilance.

**Public health implications—What are the key implications or messages for practitioners, policy makers and/or researchers in public health?**

- Practitioners and public health stakeholders should interpret these findings as hypothesis-generating reporting signals, not as evidence of incidence, absolute risk, or causality.
- Researchers should validate prioritized signals, particularly hematological and selected neurological Preferred Term-level findings, using denominator-linked epidemiological studies, active surveillance systems, and clinical adjudication.

**Abstract:**

Background/Objectives: Autoimmune and immune-mediated adverse events coded in reports listing COVID-19 vaccines as suspect products are rare, heterogeneous, and difficult to evaluate in spontaneous reporting systems. We characterized autoimmune reporting signals using strict case definitions and an active-comparator disproportionality design. Methods: FAERS reports from 2021Q1 to 2023Q4 were deduplicated and harmonized by vaccine product, platform, sex, age group, and MedDRA Preferred Term (PT). COVID-19 vaccine reports recorded as primary or secondary suspect products were compared with traditional non-COVID vaccine reports. Strict autoimmune events were grouped into neurological, endocrine, rheumatological, hematological, dermatological, and cardiac categories. Reporting odds ratios (RORs), proportional reporting ratios (PRRs), PT-level rankings, masking audits, and sensitivity analyses were evaluated. Results: The primary analysis included 4191 COVID-19 vaccine reports, 2817 comparator reports, and 311 strict-autoimmune reports contributing 316 report-category observations. Positive category-level signals were identified for dermatological (ROR 10.49; 95% CI: 2.51–43.86), endocrine (ROR 5.39; 95% CI: 1.24–23.48), hematological (ROR 4.68; 95% CI: 2.32–9.44), and neurological events (ROR 1.63; 95% CI: 1.16–2.29). Rheumatological reporting was lower, strict cardiac events were absent, and hematological signals were most consistent across sensitivity analyses. Among five adjusted-positive finite PT signals, immune thrombocytopenia was more stable, multiple sclerosis intermediate, and acquired hemophilia, myasthenia gravis, and optic neuritis sparse/imprecise. A post hoc audit identified four potential clusters among 20 acquired-hemophilia reports; cluster collapse reduced the ROR to 2.70 (95% CI 0.30–24.18; *p* = 0.654). Conclusions: These findings are hypothesis-generating and do not establish incidence, absolute risk, or causality.

## 1. Introduction

The rapid development and global deployment of COVID-19 vaccines represented one of the most important public health interventions during the SARS-CoV-2 pandemic. Large clinical trials and subsequent real-world studies demonstrated that COVID-19 vaccines substantially reduced severe disease, hospitalization, and mortality. However, because rare, delayed, or subgroup-specific adverse events may not be fully captured before authorization, continuous post-marketing vaccine safety surveillance remains essential [1,2,3].

During the COVID-19 vaccination campaign, vaccine safety monitoring relied on multiple complementary systems, including passive spontaneous reporting systems, active surveillance networks, electronic health-record databases, and international pharmacovigilance platforms. Spontaneous reporting databases such as FAERS, VAERS, EudraVigilance, VigiAccess, and VigiBase have been widely used to identify potential vaccine-safety signals. These systems are particularly useful for early signal detection, although their findings require cautious interpretation because reported events do not by themselves establish causality [2,3,4,5].

Autoimmune and immune-mediated events reported in vaccine safety surveillance have attracted scientific and regulatory attention because they may be rare, clinically heterogeneous, and difficult to distinguish from background autoimmune disease incidence. Several immune-mediated conditions, including Guillain–Barré syndrome, immune thrombocytopenia, myocarditis/pericarditis, thyroiditis, inflammatory bowel disease, rheumatologic disorders, and demyelinating events, have been evaluated in the context of COVID-19 vaccine safety surveillance [6,7,8,9].

Previous studies have assessed autoimmune and autoinflammatory reporting patterns for COVID-19 vaccines using VAERS, VigiBase, EudraVigilance, and large linked healthcare databases. For example, VAERS-based analyses have described autoimmune and autoinflammatory events across several clinical classes and stratified them by age, sex, and vaccine manufacturer [6]. VigiBase disproportionality studies have also reported potential autoimmune safety signals for selected conditions, including inflammatory bowel disease, thyroiditis, polymyalgia rheumatica, and spondyloarthritis-related terms [7]. Large multinational cohort studies have further evaluated adverse events of special interest across neurological, hematological, and cardiac outcomes, providing important epidemiological context beyond spontaneous reporting data [8].

Despite their value for early signal detection, spontaneous reporting systems have important methodological limitations. These include underreporting, stimulated reporting, incomplete clinical information, duplicate submissions, missing denominator data, and confounding by age, sex, comorbidities, infection history, and reporting behavior [2,4,10]. Therefore, disproportionality estimates should be interpreted as indicators of disproportionate reporting rather than measures of incidence, absolute risk, or causal association.

Autoimmune safety surveillance presents additional challenges. First, autoimmune and immune-mediated conditions are clinically diverse and may be coded using different MedDRA Preferred Terms. Second, broad event categories may combine clinically distinct conditions, potentially diluting strong Preferred Term-level signals. Third, non-specific immune-related terms may introduce misclassification and weaken the interpretability of category-level findings. These issues can lead to signal masking, in which a clinically meaningful signal at the Preferred Term level is obscured when events are analyzed only within broad autoimmune categories [10,11,12].

Previous pharmacovigilance studies have evaluated COVID-19 vaccine adverse-event reporting patterns using international spontaneous reporting systems, including FAERS, VigiAccess, EudraVigilance, VigiBase, and VAERS. However, most studies have focused either on overall adverse-event profiles, selected adverse events of special interest, or non-FAERS datasets. As a result, uncertainty remains regarding whether autoimmune safety signals may be diluted or masked when broad event definitions are used without systematic Preferred Term-level assessment.

In this context, FAERS was used as a complementary pharmacovigilance source rather than as a substitute for vaccine-specific passive surveillance systems or denominator-linked epidemiological databases. Its structure allows COVID-19 vaccine reports and traditional non-COVID vaccine reports to be evaluated within the same spontaneous reporting environment, supporting an active-comparator disproportionality framework. This design helps reduce some bias that may arise when vaccine reports are compared with the entire background of non-vaccine drug reports, although it does not overcome the inherent limitations of spontaneous reporting, including missing exposure denominators, reporting bias, incomplete clinical information, and lack of causal adjudication [2,3,4,5,10].

A refined FAERS-based disproportionality analysis may help address this gap by applying stricter autoimmune event definitions, harmonizing vaccine product names, classifying reports by vaccine platform, and comparing both category-level and Preferred Term-level results. Such an approach may improve the clinical specificity of signal detection and clarify whether observed disproportionality is consistent across vaccine platforms, sex strata, and age groups.

This study aimed to evaluate autoimmune and immune-mediated adverse event reporting patterns in FAERS reports listing COVID-19 vaccines as suspect products using a refined disproportionality framework. Specifically, we aimed to identify category-level autoimmune reporting signals in the COVID-19 vaccine report cohort; compare reporting patterns across mRNA, viral vector, and protein subunit vaccine platforms; assess sex- and age-stratified differences in autoimmune disproportionality; perform Preferred Term-level signal detection to identify top-ranked autoimmune safety signals; and evaluate potential signal masking, misclassification, and robustness through sensitivity analyses.

## 2. Materials and Methods

### 2.1. Study Design and Data Source

This study was designed as a retrospective pharmacovigilance disproportionality analysis of spontaneous adverse event reports from the U.S. Food and Drug Administration Adverse Event Reporting System (FAERS). The analysis evaluated autoimmune and immune-mediated reporting patterns in FAERS reports listing COVID-19 vaccines as suspect products and examined whether these patterns differed by vaccine platform, product group, sex, age group, and individual MedDRA Preferred Term (PT). Quarterly public FAERS files from 2021Q1 through 2023Q4 were used. Adverse-event terms were analyzed as MedDRA Preferred Terms (PTs) as coded in the FAERS quarterly REAC files. FAERS was selected to enable comparison of COVID-19 vaccine reports with traditional vaccine reports within a single spontaneous reporting database, thereby supporting an internally consistent active-comparator disproportionality design. The autoimmune PT dictionary was harmonized against MedDRA version 26.1 before analysis. The analysis was conducted at the report-category or PT level for autoimmune event summaries, as appropriate. Because FAERS is a passive spontaneous reporting system, all results were interpreted as disproportionality signals for hypothesis generation, not as incidence estimates, absolute risks, or evidence of causality.

### 2.2. Data Extraction, Deduplication, and Report Harmonization

Quarterly public FAERS files covering 2021Q1–2023Q4 were processed from the DEMO, DRUG, REAC, and OUTC, and THER tables. The DEMO table served as the report-level backbone and provided CASEID, PRIMARYID, FDA receipt date (FDA_DT), age, sex, and reporting-period information; DRUG provided product names and drug-role codes; REAC provided MedDRA Preferred Terms; and OUTC provided seriousness outcomes. Deterministic deduplication was performed at the CASEID level before cohort construction. For each CASEID, only the latest case version was retained, defined by the most recent FDA_DT; when two or more versions had the same FDA_DT, the version with the highest PRIMARYID was retained. All DRUG, REAC, and OUTC records linked to the retained PRIMARYID were then analyzed together. Accordingly, the unit of analysis was one deduplicated FAERS report/case, not an individual drug record. Product names, adverse-event terms, demographic variables, drug-role codes, and outcome codes were harmonized after deduplication. Seriousness was defined using FAERS OUTC codes; a report was classified as serious if death, a life-threatening event, hospitalization, disability, congenital anomaly, required intervention, or another serious outcome was recorded.

Patient age and sex were standardized before subgroup analyses. Age was retained as reported when interpretable and was grouped into predefined categories only after cohort construction. Reports with missing, unknown, or non-classifiable age or sex were included in descriptive summaries but excluded from the corresponding stratified disproportionality comparisons. Seriousness outcomes were summarized both as a mutually exclusive binary serious/non-serious indicator and as individual OUTC code frequencies, recognizing that a single FAERS report may contain more than one seriousness outcome code. These preprocessing rules were applied consistently to COVID-19 vaccine and comparator cohorts. Cohort-level age and sex distributions, including missing or unknown values, were summarized separately for the complete COVID-19 vaccine and comparator cohorts. Percentages used the full cohort denominators; no demographic values were imputed.

For time-to-onset analysis, EVENT_DT from DEMO was linked to qualifying PS/SS COVID-19 vaccine START_DT values in THER using retained PRIMARYID and DRUG/THER sequence numbers. Only complete YYYYMMDD dates were used; partial dates were not imputed. When multiple valid START_DT values were available, the latest date on or before EVENT_DT was selected. Time to onset was calculated as EVENT_DT minus START_DT; negative intervals were invalid, and intervals >365 days were reported separately and excluded from the 0–365-day analysis.

### 2.3. Identification of COVID-19 Vaccine Reports and Platform Assignment

COVID-19 vaccine reports were identified using the following prespecified standardized product terms: TOZINAMERAN, ELASOMERAN, VAXZEVRIA, NUVAXOVID, COVISHIELD, AD26.COV2, SPIKEVAX, COMIRNATY, BNT162B2, and MRNA-1273. A report entered the primary COVID-19 vaccine cohort when at least one of these terms occurred in a DRUG record coded as primary suspect (PS) or secondary suspect (SS). Bare manufacturer names or manufacturer-only tokens were not used for cohort assignment. A more restrictive PS-only cohort was constructed for sensitivity analysis. The complete exposure-identification dictionary is provided in Supplementary Table S12.

PS + SS was used for the primary analysis because both are reporter-assigned FAERS suspect roles and inclusion of SS reports improves sensitivity when a vaccine is coded as secondary suspect but is not designated as the principal suspect in a report involving multiple products. The same definition was applied to both cohorts, while a PS-only sensitivity analysis assessed potential attribution bias from co-reported drugs.

Throughout, “COVID-19 vaccine report” refers to a deduplicated FAERS report listing a prespecified COVID-19 vaccine as a PS or SS product. This term describes report content only; exposure before event onset was not consistently confirmed, and no clinical adjudication or causal attribution was performed. Reported events therefore do not represent confirmed vaccine-caused outcomes.

For product and platform summaries, TOZINAMERAN, BNT162B2, and COMIRNATY were mapped to Pfizer-BioNTech (Pfizer Inc., New York, NY, USA; BioNTech SE, Mainz, Germany); ELASOMERAN, MRNA-1273, and SPIKEVAX to Moderna (Moderna, Inc., Cambridge, MA, USA); VAXZEVRIA and COVISHIELD to AstraZeneca (AstraZeneca plc, Cambridge, UK); AD26.COV2 to Janssen (Janssen Biotech, Inc., Horsham, PA, USA); and NUVAXOVID to Novavax (Novavax, Inc., Gaithersburg, MD, USA). These product mappings were used only after report-level cohort assignment. Reports containing more than one COVID-19 vaccine term were counted once in the overall COVID-19 cohort and were assigned using mutually exclusive product/platform rules, including a separate multiple-COVID-product group when appropriate, so they were not double-counted in product or platform denominators.

### 2.4. Autoimmune Event Definition and MedDRA Grouping

Because the boundary between classical autoimmune disease, immune-mediated inflammatory disease, and autoinflammatory disease is not always clear, the strict dictionary included clinically specific autoimmune and immune-mediated Preferred Terms, whereas non-specific inflammatory or organ-level terms were reserved for sensitivity analyses. Autoimmune and immune-mediated adverse events were identified at the MedDRA PT level. The primary strict autoimmune dictionary included 48 MedDRA Preferred Terms. Fourteen additional broad-only Preferred Terms were prespecified for sensitivity analyses but were not included in the primary strict definition (Supplementary Table S1). A strict autoimmune dictionary was applied to improve clinical specificity and to avoid inclusion of vague or non-specific immune-related reactions. Selected PTs were grouped into six clinically predefined categories: neurological, endocrine, rheumatological, hematological, dermatological, and cardiac autoimmune events. A single report could contribute to more than one autoimmune category if it contained PTs belonging to different categories; therefore, unique reports and report-category observations were summarized separately. The final strict dictionary included 48 PTs. Fourteen additional broad-only PTs were reserved for sensitivity analyses and were not included in the primary strict autoimmune definition. Basedow’s disease and Graves’ disease were retained as separate PTs; no CASEID contained both, and category-level collapsing prevented double-counting.

### 2.5. Comparator Definition

Traditional non-COVID vaccine reports were used as the active comparator to reduce bias that could arise from comparison with all non-vaccine drug reports. The main comparator comprised deduplicated reports containing a traditional vaccine in a PS or SS DRUG record, identified by the generic substrings VACCINE, VAX, or TOXOID and/or by a prespecified curated list of 43 traditional-vaccine brand names. Any report containing one of the prespecified COVID-19 vaccine terms was excluded from the comparator, even when a traditional vaccine was coadministered. Additional sensitivity comparators included a PS-only cohort and a brand-only cohort restricted to the curated traditional-vaccine brand list. The complete comparator-identification dictionary is provided in Supplementary Table S12.

Cohort assignment and event counting were performed at the deduplicated-report level. A report containing multiple COVID-19 vaccine names contributed once to the overall COVID-19 cohort. Reports containing both COVID-19 and non-COVID vaccines were assigned to the COVID-19 cohort and excluded from the comparator. Within autoimmune analyses, multiple PTs belonging to the same predefined autoimmune category were collapsed so that a report contributed no more than one observation to that category; however, a report containing PTs from different autoimmune categories could contribute one observation to each relevant category. Consequently, unique strict-autoimmune reports and report-category observations were reported separately.

A reviewer-requested product-level audit normalized PS/SS DRUGNAME values and mapped exact tokens to harmonized vaccine products and classes. Reports were assigned to a single class or a multiple-class group and counted once in the pooled comparator, while nonexclusive counts described co-reported vaccines. Non-vaccine, generic, therapeutic, experimental, unclassifiable, and punctuation-variant COVID-19 matches were excluded from the validated-comparator sensitivity analysis.

A reviewer-requested exact-token audit excluded 206 of 2817 protocol-screened comparator reports: 147 non-vaccine substring matches, 47 unclassifiable unspecified-vaccine reports, 10 therapeutic or experimental vaccine terms, and two punctuation-variant COVID-19 matches, yielding a product-classifiable comparator of 2611 reports. Because the 2817-report cohort was prespecified and the audit was post hoc, it remained the primary comparator, while the 2611-report cohort was used as a stringent sensitivity comparator.

### 2.6. Disproportionality Measures and Signal Detection

For each autoimmune category and PT, a 2 × 2 table was constructed: a, COVID-19 vaccine reports with the event; b, COVID-19 vaccine reports without the event; c, comparator reports with the event; and d, comparator reports without the event. The reporting odds ratio (ROR) was calculated as (a/b)/(c/d), and the proportional reporting ratio (PRR) as [a/(a + b)]/[c/(c + d)]. For estimable tables, two-sided 95% confidence intervals were calculated on the log scale using Wald formulas: exp[ln(ROR) ± 1.96 × √(1/a + 1/b + 1/c + 1/d)] and exp[ln(PRR) ± 1.96 × √(1/a − 1/(a + b) + 1/c − 1/(c + d))].

Two-sided Fisher exact *p* values were calculated for all non-degenerate 2 × 2 tables using scipy.stats.fisher_exact(…, alternative = ‘two-sided’). Fisher testing was applied uniformly to PT-level, category-level, subgroup, product-specific, comparator, and sensitivity analyses to avoid data-dependent test selection.

Benjamini–Hochberg adjustment was performed within prespecified families: five primary category-level tests; 27 strict PT-level tests; 10 pooled platform-category tests; five within-platform tests per platform; five product-specific tests per product; 10 sex-stratified tests; and 14 age-stratified tests. Each sensitivity scenario, validated-comparator analysis, and comparator-class analysis was adjusted separately. Interaction analyses retained their prespecified Wald procedures across five platforms, four sex, 10 age-pair, and three age-trend tests. Product-, class-, sensitivity-, and interaction analyses were secondary or exploratory.

Primary ROR and PRR estimates were calculated from observed counts without pseudo-counts. If either event cell (a or c) was zero, ROR/PRR estimates and confidence intervals were reported as N/E, although Fisher’s exact test was retained for non-degenerate tables. If a = c = 0, no test was performed. Benjamini–Hochberg-supported zero-cell results were described as adjusted exact imbalances and excluded from finite-ROR rankings. The Haldane–Anscombe correction was used only for explicitly labeled continuity-corrected sensitivity estimates, and the former Yates-corrected results were retained only in Supplementary Table S20.

A finite positive signal required ROR and PRR estimates above 1.0, lower 95% confidence bounds above 1.0, and a Fisher–Benjamini–Hochberg-adjusted *p* value below 0.05. Lower-reporting findings required estimates below 1.0, upper confidence bounds below 1.0 and adjusted statistical support. Zero-cell findings with adjusted Fisher support were reported separately as exact imbalances. Interpretation also considered ROR/PRR concordance, interval precision, and the stability of the underlying counts.

### 2.7. Subgroup, PT-Level, and Case-Definition Sensitivity Analyses

Category-level analyses were performed for neurological, endocrine, rheumatological, hematological, dermatological, and cardiac autoimmune events. Subgroup analyses were conducted by vaccine platform, product group, sex, and age group. Sex-stratified analyses included female and male reports; reports with unknown sex were retained in descriptive summaries but excluded from the primary female-versus-male comparison. Age groups were predefined as <40 years, 40–64 years, and ≥65 years; reports with missing or non-classifiable age were retained descriptively but excluded from age-stratified disproportionality calculations.

Formal subgroup heterogeneity was assessed using ratios of RORs (RORR = ROR_1_/ROR_2_), rather than differences in stratum-specific significance. Sex- and age-specific RORRs were evaluated on the log scale using two-sided Wald 95% CIs and *p* values, with variance derived from both stratum-specific log-RORs. For platform comparisons sharing the same comparator, comparator terms canceled, and variance was based on platform event and non-event counts. Ordered age trends were tested by inverse-variance-weighted regression across age scores 0, 1, and 2. Uncorrected estimates required nonzero cells. Benjamini–Hochberg adjustment was applied separately to 5 platform, 4 sex, 10 age-pair, and 3 age-trend tests. All interaction analyses were exploratory; significance in one subgroup alone was not considered evidence of effect modification.

PT-level disproportionality analysis was used to determine whether clinically specific PT patterns were attenuated or obscured after aggregation into broader categories. PTs were ranked by finite ROR magnitude while considering event counts, interval precision, and adjusted statistical support. Category- and PT-level findings were compared for concordance and discordance. Aggregation-related dilution was defined descriptively as attenuation or directional change after category aggregation or inclusion of broad, non-specific PTs; it was not treated as formal drug–drug masking.

Because acquired hemophilia had the highest finite PT-level ROR, a post hoc independence audit reviewed CASEID/PRIMARYID histories and key report identifiers. Distinct CASEIDs with shared authorization numbers or highly concordant clinical and vaccine profiles were flagged as potential parallel submissions. Potential clinical-episode clusters were then collapsed in an exploratory analysis using an unadjusted two-sided Fisher exact test.

### 2.8. Sensitivity Analyses, Software, and Ethics

Sensitivity analyses repeated the primary approach under alternative assumptions: broad PT definitions, peak-period restriction, brand-only comparator definition, and primary-suspect-only restriction. These analyses evaluated the robustness of signals to event definition, comparator selection, reporting period, and drug-role assignment. The peak-period analysis restricted the dataset to the period of highest COVID-19 vaccine reporting intensity (January 2021 through June 2022), whereas the primary-suspect-only analysis assessed whether results persisted under a more restrictive suspect-role definition. To evaluate the cohort-level imbalance in seriousness coding, an additional sensitivity analysis restricted both cohorts to reports containing at least one serious FAERS OUTC code (death, life-threatening outcome, hospitalization, disability, congenital anomaly, or other serious outcome). The same strict PT dictionary, category-counting rules, ROR/PRR calculations, confidence intervals, and within-table Benjamini–Hochberg adjustment were then reapplied. All analyses were performed in Python 3.13.13 using pandas 3.0.2, NumPy 2.4.4, SciPy 1.17.1, statsmodels 0.14.6, and openpyxl 3.1.5. The study used publicly available, de-identified FAERS data; therefore, institutional review board approval and informed consent were not required.

Additional reviewer-requested sensitivity analyses compared the COVID-19 vaccine cohort separately with the largest mutually exclusive traditional-vaccine classes, defined as those with ≥100 single-class comparator reports. Estimates based on <5 comparator event reports were considered sparse and interpreted descriptively. Haldane–Anscombe 0.5 continuity-corrected estimates were reported separately for zero-cell and sparse comparisons.

To assess the influence of differential seriousness reporting between cohorts, category-level disproportionality was repeated after restricting both cohorts to reports containing at least one serious FAERS OUTC code (DE, LT, HO, DS, CA, RI, or OT). Category-specific 2 × 2 tables were reconstructed within the serious COVID-19 vaccine cohort (*N* = 3757) and serious comparator-vaccine cohort (*N* = 2105). RORs, PRRs, log-Wald 95% confidence intervals, two-sided Fisher exact *p* values, zero-cell handling, and Benjamini–Hochberg adjustment were applied as in the primary analysis.

### 2.9. Evidence Robustness Classification

To distinguish effect-size magnitude from evidentiary support, a reviewer-requested post hoc descriptive robustness classification was applied separately from statistical signal detection. Finite estimates were classified as having limited support when either event count (a or c) was <5 or the ROR 95% CI fold-width, calculated as the upper divided by the lower confidence limit, exceeded 10. Estimates were classified as more stable when both event counts were ≥5, the CI fold-width was ≤10, and the parent category retained an adjusted result in the same direction as the primary analysis in at least three of five sensitivity analyses (broad PT, peak-window, brand-only comparator, PS-only, and serious-report-only). All remaining finite estimates were classified as intermediate. For PT-level findings, sensitivity consistency referred to the parent category and was not interpreted as PT-specific replication. Zero-cell findings remained a separate non-estimable class. This framework was descriptive and did not constitute formal certainty-of-evidence grading. PTs were ordered by finite ROR magnitude for presentation only; rank did not indicate evidentiary strength. A more stable classification indicates support across the defined domains but does not require concordance in every sensitivity scenario; directional reversals were therefore reported explicitly.

## 3. Results

### 3.1. Analysis Cohorts and Descriptive Profile

From 2021Q1 through 2023Q4, deterministic CASEID-level deduplication yielded 4,707,736 FAERS reports. PS/SS exposure screening identified 4191 COVID-19 vaccine reports and 2817 traditional non-COVID vaccine comparator reports (Supplementary Figure S1 and Table S2). Sensitivity cohorts included a COVID-19 PS-only cohort (*n* = 735), a traditional-vaccine PS-only comparator (*n* = 485), a brand-only traditional-vaccine comparator (*n* = 1355), and a peak-period analytic cohort (January 2021 through June 2022) comprising 2520 COVID-19 vaccine reports and 1481 comparator reports (Tables S3–S5 and S11).

The comparator cohort was heterogeneous, led by influenza (684/2817; 24.3%), meningococcal (318; 11.3%), herpes zoster (289; 10.3%), pneumococcal (281; 10.0%), BCG/tuberculosis (139; 4.9%), and tetanus/diphtheria/pertussis-containing vaccines (123; 4.4%); 298 reports (10.6%) involved multiple classes. Exact-token validation excluded 206 reports—147 non-vaccine matches, 47 unclassifiable unspecified-vaccine reports, 10 therapeutic/experimental terms, and two COVID-19 leakage records—yielding 2611 product-classifiable reports (Table S13).

The exposure cohorts differed demographically (Table 1, Panel C). Among reports with known age, median age was 61 years (IQR 46–73) in the COVID-19 vaccine cohort and 55 years (IQR 11–70) in the comparator cohort. Age was unknown in 13.8% versus 35.3%, and sex in 8.4% versus 20.1%; females comprised 56.5% and 48.8%, respectively. Consequently, age- and sex-stratified analyses retained fewer comparator reports, which was considered when interpreting subgroup estimates.

Application of the strict autoimmune PT dictionary identified 311 unique reports listing a COVID-19 vaccine as a PS or SS product. Because multiple PTs within the same category were counted once, whereas PTs from different categories were retained separately, these 311 reports produced 316 report-category observations (Supplementary Figure S1). Neurological events were the largest category (*n* = 115), followed by rheumatological (*n* = 92), hematological (*n* = 62), dermatological (*n* = 31), and endocrine events (*n* = 16); no strict cardiac autoimmune observations were identified.

The median reported subject age in the strict-autoimmune subset was 59.0 years (interquartile range, 44.25–68.00). Female sex was recorded in 181 reports (58.2%), male sex in 105 reports (33.8%), and sex was unknown in 25 reports (8.0%). Endocrine events had the youngest median age (30.0 years) and the strongest female predominance (14/16; 87.5%), whereas hematological and dermatological events were coded in reports involving older subjects and showed more balanced sex distributions. Most strict-autoimmune reports were serious: 310 of 311 reports (99.7%) had at least one serious FAERS OUTC code, most commonly other serious outcome or hospitalization. At the full-cohort level, 3757 of 4191 COVID-19 vaccine reports (89.6%) and 2105 of 2817 traditional-vaccine comparator reports (74.7%) were coded as serious, an absolute difference of 14.9 percentage points (Table S2).

COVID-19 vaccine product distribution was dominated by Pfizer-BioNTech reports (3431/4191; 81.9%), followed by AstraZeneca (367; 8.8%), Moderna (291; 6.9%), Janssen (17; 0.4%), and multiple COVID-19 vaccine brands (85; 2.0%). Among strict autoimmune cases, Pfizer-BioNTech accounted for 246 cases (79.1%), AstraZeneca for 32 (10.3%), Moderna for 21 (6.8%), and multiple-brand reports for 12 (3.9%). After correction for overlapping product terms, the platform denominators were 3807 mRNA vaccine reports and 384 viral-vector vaccine reports; no protein-subunit vaccine reports were present in the corrected platform-specific dataset (Table 1).

### 3.2. Category-Level Autoimmune Disproportionality Signals

In the primary active-comparator analysis, dermatological, endocrine, hematological, and neurological categories showed positive disproportionality (Table 2), with Fisher–BH-adjusted *p* values of 4.42 × 10^−5^, 0.0138, 2.73 × 10^−6^, and 0.00572, respectively. Estimates were highest for dermatological (31 vs. 2; ROR 10.49, 95% CI 2.51–43.86), followed by endocrine (16 vs. 2; ROR 5.39, 95% CI 1.24–23.48) and hematological events (62 vs. 9; ROR 4.68, 95% CI 2.32–9.44). Neurological events were most frequent (115 vs. 48) but showed a smaller elevation (ROR 1.63, 95% CI 1.16–2.29). PRR estimates were concordant with the RORs.

Under the robustness framework, the hematological positive signal and the lower rheumatological estimate were classified as more stable. The neurological positive signal was intermediate because it was not consistently retained across sensitivity analyses. Dermatological and endocrine category estimates were sparse/imprecise because each relied on only two comparator events and had wide confidence intervals. Thus, their larger point estimates did not represent stronger evidence.

Rheumatological events were frequent but did not show increased reporting relative to the active comparator (92 vs. 102; ROR 0.60, 95% CI 0.45–0.80; PRR 0.61, 95% CI 0.46–0.80). Thus, this category was interpreted as reduced reporting compared with traditional vaccines rather than as a positive autoimmune signal. Strict cardiac autoimmune events were absent in both cohorts and were therefore not estimable.

### 3.3. Case-Definition Sensitivity and Broad-Term Analysis

Comparison of strict and broad definitions showed that the main source of sensitivity was inclusion of broad, non-specific PTs rather than overlap between strict categories. Neurological and hematological estimates attenuated under the broad definition, while dermatological reporting no longer met the adjusted-positive criterion.

Broad cardiac terms produced a signal despite the absence of strict autoimmune cardiac events. Thus, broader PT sets may either dilute clinically specific reporting patterns or generate non-specific aggregate findings, depending on their relative distribution across cohorts.

### 3.4. Platform- and Product-Specific Patterns

Platform-specific analyses used corrected mutually exclusive platform denominators (Table 3). In the mRNA stratum, positive signals were identified for neurological (ROR 1.72, 95% CI 1.22–2.42), hematological (ROR 4.40, 95% CI 2.17–8.94), and dermatological events (ROR 10.80, 95% CI 2.58–45.32). Rheumatological events were lower than comparator vaccines (ROR 0.57, 95% CI 0.42–0.77), endocrine events were elevated, but not adjusted-positive, and cardiac events were not estimable. In the viral-vector stratum, endocrine (ROR 18.57, 95% CI 3.59–96.05) and hematological events (ROR 7.49, 95% CI 2.95–18.98) were signal-positive. Dermatological events had an elevated but non-adjusted-positive estimate, while neurological and rheumatological events showed no positive signal. Hematological events were the only category with positive signals in both mRNA and viral-vector strata. Protein-subunit vaccines could not be assessed because no eligible reports were present in the corrected platform dataset.

Formal platform interaction was supported only for endocrine events (mRNA-to-viral-vector ROR 0.22, 95% CI 0.08–0.64; *p* = 0.0052; BH-adjusted *p* = 0.0258), indicating greater relative endocrine reporting in the viral-vector stratum. No adjusted interaction was detected for neurological, rheumatological, hematological, or dermatological events (all BH-adjusted *p* ≥ 0.193). Thus, differences in within-platform signals were not interpreted as general platform heterogeneity (Table S17).

Product-specific patterns broadly matched platform-level results but were limited by sparse data (Table S6). Pfizer-BioNTech showed Fisher–BH-supported dermatological, hematological, neurological, and endocrine findings, although the endocrine estimate remained sparse/imprecise (11 vs. 2; adjusted *p* = 0.0470). Moderna’s clearest signal was hematological, whereas AstraZeneca/Covishield showed endocrine and hematological disproportionality; Janssen and Novavax were not meaningfully evaluable.

### 3.5. Sex- and Age-Stratified Analyses

Sex-stratified Fisher analysis showed adjusted-positive hematological and dermatological findings in both sexes (Table 4). Hematological RORs were 5.66 in females (95% CI 1.72–18.62; BH *p* = 0.00353) and 4.55 in males (1.60–12.95; *p* = 0.00383). Dermatological estimates were also positive but sparse/imprecise because each comparator stratum contained one event (female ROR 8.16; *p* = 0.0357; male ROR 7.82; *p* = 0.0387). Neurological reporting was additionally positive in females, whereas endocrine reporting was not; rheumatological reporting was lower only in females.

Formal sex interaction was supported only for rheumatological events (female-to-male ROR 0.30, 95% CI 0.14–0.64; *p* = 0.0018; BH-adjusted *p* = 0.0070). No interaction was detected for neurological, hematological, or dermatological events (all BH-adjusted *p* ≥ 0.491); endocrine and cardiac comparisons were not estimable. These exploratory findings did not support sex differences in the principal positive patterns (Table S17).

Age-stratified analysis identified different patterns across predefined age groups (Table 5). Among individuals aged <40 years, neurological events were signal-positive (ROR 3.26, 95% CI 1.45–7.35; Fisher–BH *p* = 0.0108), and endocrine events showed an adjusted exact higher-reporting imbalance based on nine versus zero reports (Fisher–BH *p* = 0.0056), with ROR and PRR remaining non-estimable. In the 40–64-year stratum, no category showed an adjusted finite positive signal; rheumatological reporting was lower relative to comparator vaccines (Fisher–BH *p* = 5.00 × 10^−4^). Among individuals aged ≥65 years, hematological events were signal-positive (ROR 7.84, 95% CI 1.88–32.71; Fisher–BH *p* = 0.0026), neurological reporting was lower than comparator vaccines (Fisher–BH *p* = 0.0130), and dermatological events were adjusted-positive but sparse/imprecise (ROR 7.76, 95% CI 1.03–58.42; Fisher–BH *p* = 0.0441). These findings indicate that the strongest age-specific finite positive signals were neurological events in younger adults and hematological and dermatological events in older adults.

Formal testing supported an age interaction only for neurological events: the <40-to-≥65 ROR was 12.53 (95% CI 3.63–43.29; *p* = 6.39 × 10^−5^; BH-adjusted *p* = 0.00064), the ≥65-to-40–64 ratio was 0.19 (0.06–0.57; *p* = 0.0029; BH-adjusted *p* = 0.0146), and the ordered trend was significant (BH-adjusted *p* = 0.00026). The <40-to-40–64 comparison was not significant. The nominal hematological interaction did not survive correction, and all other age interactions were non-significant or non-estimable (Table 5 and Table S7).

### 3.6. Preferred Term-Level Signals

Fisher–Benjamini–Hochberg analysis of 27 non-degenerate strict PT tables retained five finite adjusted-positive findings: acquired hemophilia (BH *p* = 0.00316), myasthenia gravis (0.0117), optic neuritis (0.0117), immune thrombocytopenia (0.00879), and multiple sclerosis (0.00181) (Table 6 and Table S8). Immune thrombocytopenia (37 vs. 8; ROR 3.13, 95% CI 1.45–6.73) was classified as more stable, multiple sclerosis as intermediate, and the other three as sparse/imprecise. Exact testing also identified higher reporting of Miller Fisher syndrome (9 vs. 0; BH *p* = 0.0369), lower reporting of acute disseminated encephalomyelitis (0 vs. 8; *p* = 0.00366), and lower finite reporting of juvenile idiopathic arthritis (1 vs. 6; *p* = 0.0474). Zero-cell PTs remained non-estimable and were excluded from finite-ROR rankings. The Yates sensitivity analysis retained the same five finite positive PTs (Table S20).

The acquired-hemophilia audit showed marked clustering: 19 of 20 reports originated from France, 13 were received in 2022Q2, and 12 shared three AUTH_NUMs, suggesting four potential episodes comprising 9, 6, 4, and 1 reports. Cluster collapse reduced the ROR to 2.70 (95% CI 0.30–24.18; Fisher *p* = 0.654). The sole comparator report closely matched the six-report cluster, suggesting a possible unconfirmed cross-cohort parallel submission (Table S18).

The five adjusted-positive finite PTs should not be read as a flat set of equally supported findings. Immune thrombocytopenia had the strongest robustness profile. Multiple sclerosis had adequate counts and a comparatively narrow interval but was classified as intermediate because the neurological category was not consistently positive in sensitivity analyses. Acquired hemophilia, myasthenia gravis, and optic neuritis were sparse/imprecise because their comparator counts were one, two, and three and their intervals were wide. Dermatological and endocrine category-level signals were not matched by adjusted-positive finite PT-level signals. Zero-cell PTs were excluded from the finite ranking; Miller Fisher syndrome and acute disseminated encephalomyelitis were instead reported as adjusted exact imbalances in opposite directions (Tables S8–S10 and S16).

Table 6 should therefore be interpreted as an effect-size ranking rather than a hierarchy of evidentiary strength. The high ROR for acquired hemophilia reflects the contrast of 20 COVID-19 vaccine reports with only one comparator report, which produces a wide confidence interval and was highly sensitive to the post hoc cluster audit; after collapsing potential parallel submissions, the estimate was attenuated and no longer statistically supported. In contrast, immune thrombocytopenia and multiple sclerosis were based on larger comparator counts and narrower intervals, providing comparatively stronger support. Myasthenia gravis and optic neuritis remained adjusted-positive but sparse/imprecise, whereas the remaining ranked PTs did not represent adjusted-positive finite signals; rheumatoid arthritis showed lower reporting relative to the comparator. Thus, the table separates the magnitude of disproportionality from its precision and robustness.

### 3.7. Forest Plot of the Top 10 PT-Level Autoimmune Signals

**Figure 1:**
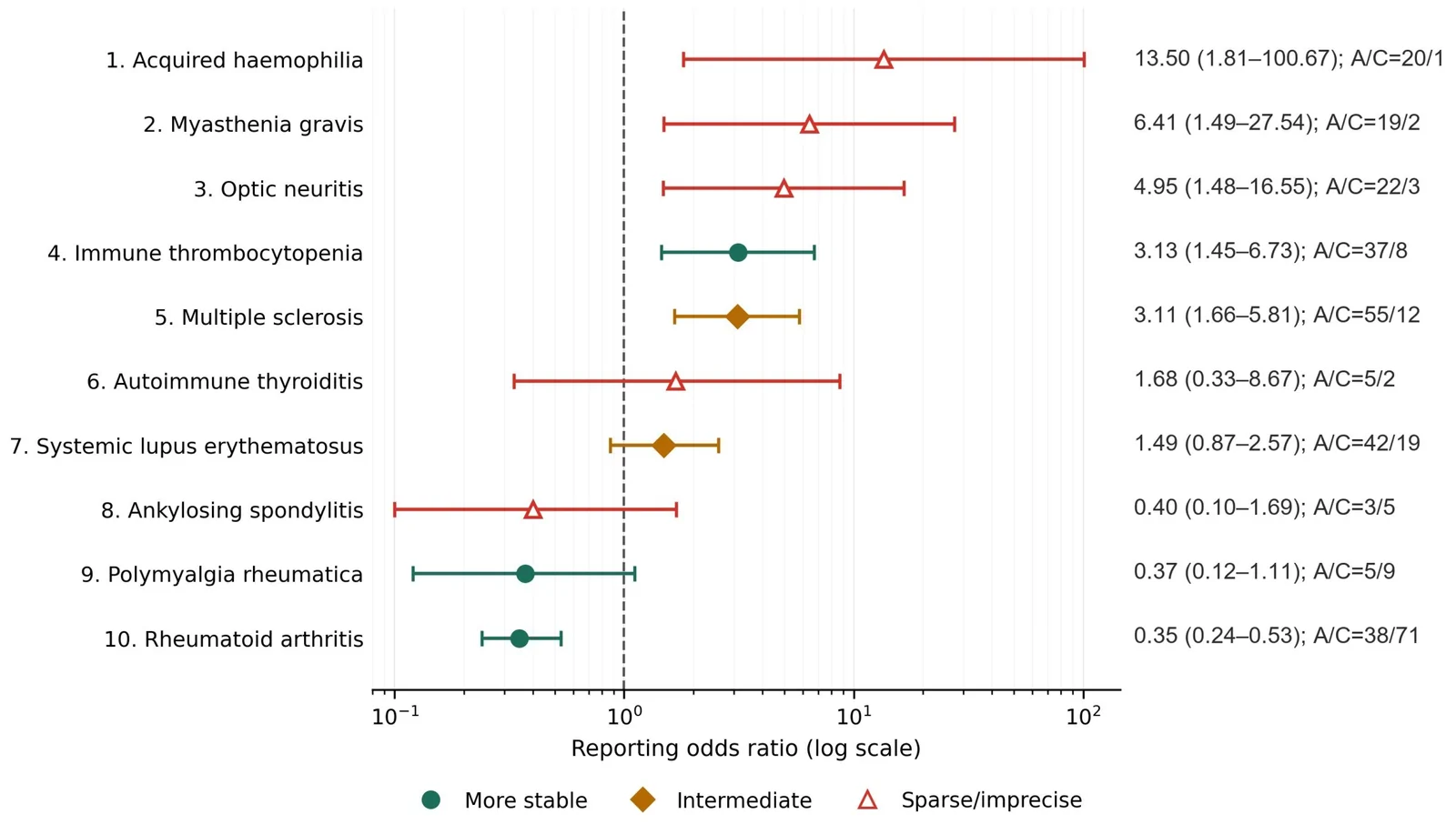
Forest plot of the top 10 autoimmune Preferred Terms ordered by finite reporting odds ratio. Error bars indicate 95% confidence intervals; labels show A/C event counts. Marker shape and color indicate the descriptive robustness tier. The vertical dashed line marks ROR = 1. Rank reflects effect-size magnitude only and does not represent evidentiary strength. Preferred Terms with zero comparator events were excluded from the finite-ROR ranking.

Figure 1 retains the finite-ROR ordering but overlays the robustness classification. The ranking is descriptive: a higher ROR does not imply stronger evidence. Acquired hemophilia remained first by ROR magnitude but was marked sparse/imprecise because C = 1 and its 95% CI ranged from 1.81 to 100.67. Its position is based on the protocol report-level estimate; the targeted cluster-collapsed analysis was not statistically supported and is reported separately in Table S18.

Among adjusted-positive PTs, immune thrombocytopenia was more stable, multiple sclerosis was intermediate, and myasthenia gravis and optic neuritis were sparse/imprecise. This ordering by evidence robustness therefore differed from the ordering by ROR magnitude.

Among the remaining ranked estimates, autoimmune thyroiditis and ankylosing spondylitis were sparse/imprecise, systemic lupus erythematosus was intermediate, and polymyalgia rheumatica and rheumatoid arthritis had more stable estimates in the lower-reporting direction. Robustness describes support and precision, not whether an estimate constitutes a positive signal.

Figure 1 provides a visual explanation of the same distinction. Horizontal position relative to ROR = 1 indicates the direction and magnitude of reporting disproportionality, whereas confidence-interval width reflects precision; the displayed A/C event counts and robustness markers indicate the amount and stability of evidence underlying each estimate. Acquired hemophilia is farthest to the right but has the widest interval and limited support because of the single comparator event and clustering. Immune thrombocytopenia and multiple sclerosis have smaller RORs but tighter intervals and comparatively stronger evidentiary support. Estimates whose confidence intervals cross 1 are not supported as finite positive signals, whereas the rheumatoid arthritis estimate lies below 1, indicating lower relative reporting. PTs with zero comparator events, such as Miller Fisher syndrome, are not shown because a finite uncorrected ROR cannot be estimated, even when Fisher exact testing identifies an adjusted exact imbalance.

### 3.8. Sensitivity Analyses

Sensitivity analyses evaluated the influence of broad PT definitions, reporting window, comparator definition, and suspect-drug role restriction (Table S11). Hematological events were the most consistent finite positive signal, remaining elevated in the primary strict analysis, broad PT sensitivity analysis, peak-window analysis (January 2021 through June 2022), and brand-only comparator analysis. Neurological events were positive in the primary strict and brand-only comparator analyses but were attenuated under the broad PT definition and lower than comparator vaccines during the peak window. Dermatological and endocrine signals were strong in the primary strict analysis but were more sensitive to zero-cell patterns and comparator definition.

The serious-report-only analysis (3757 COVID-19 and 2105 comparator reports) retained positive hematological (ROR 3.91, 95% CI 1.94–7.88; Fisher–BH *p* = 3.89 × 10^−5^) and dermatological findings (ROR 8.75, 95% CI 2.09–36.59; *p* = 0.000256) (Table S15). Neurological (ROR 1.45, 95% CI 1.02–2.05; *p* = 0.0370) and endocrine findings (ROR 4.50, 95% CI 1.03–19.58; *p* = 0.0341) were attenuated but remained adjusted-supported. Rheumatological reporting remained lower (ROR 0.50, 95% CI 0.37–0.66; *p* = 1.23 × 10^−5^), while cardiac events were not estimable.

The primary-suspect-only scenario produced sparse estimates because it included 735 COVID-19 vaccine reports and 485 comparator reports. In that scenario, rheumatological events showed a positive finite estimate (ROR 12.15, 95% CI 1.62–91.32), but this result was based on only one comparator event and should not override the primary PS + SS finding of reduced reporting relative to comparator vaccines. Overall, the sensitivity analyses supported the robustness of the hematological signal, indicated moderate sensitivity of neurological findings to analytic choices, and confirmed that broad cardiac signals reflected non-specific term inclusion rather than strict autoimmune cardiac events.

As a reviewer-requested post hoc sensitivity analysis, the comparator was restricted to 2611 product-classifiable traditional-vaccine reports; the prespecified 2817-report cohort remained primary because exact-token validation was post hoc. Category-level conclusions were unchanged: neurological ROR 1.54 (95% CI 1.09–2.17; BH *p* = 0.0154), endocrine 5.00 (1.15–21.76; *p* = 0.0154), rheumatological 0.58 (0.43–0.77; *p* = 0.000327), hematological 4.34 (2.15–8.75; *p* = 9.71 × 10^−6^), and dermatological 9.72 (2.32–40.65; *p* = 9.67 × 10^−5^). Class-specific analyses remained exploratory because comparator-event counts were often sparse or zero (Table S14).

Suspect-role composition was nearly identical between cohorts: 735/4191 COVID-19 reports (17.5%) and 485/2817 comparator reports (17.2%) contained a qualifying vaccine coded as PS, whereas 82.5% and 82.8%, respectively, were SS-only. These mutually exclusive counts indicate that PS-only restriction reduced both cohorts by approximately 83%, resulting in sparse tables.

In the primary PS + SS analysis, adjusted positive signals were observed for neurological, endocrine, hematological, and dermatological events, while rheumatological reporting was lower than in comparators (ROR 0.60, 95% CI 0.45–0.80). Under PS-only restriction, only neurological and rheumatological estimates remained calculable without continuity correction. The neurological estimate attenuated to 1.16 (95% CI 0.34–3.97), whereas the rheumatological estimate reversed direction (ROR 12.15, 95% CI 1.62–91.32; Fisher–Benjamini–Hochberg *p* = 0.00686), based on 18 versus one event. Other categories remained non-estimable because of zero cells and did not show adjusted exact imbalances. Thus, the rheumatological result was sensitive to suspect-role classification, but the single comparator event and wide confidence interval preclude robust interpretation (Table S11).

### 3.9. Descriptive Time to Onset and Temporal Data Completeness

Among 311 strict-autoimmune COVID-19 vaccine reports, 202 (65.0%) had exact linked vaccination and event dates. Of these, 134 yielded nonnegative intervals; excluding two intervals > 365 days left 132 reports (42.4%) for analysis, while 68 (21.9%) were chronologically invalid (Table S19).

Median time to onset was 30 days (IQR 10.5–74.0). Category-specific medians were 13 days for neurological, 5.5 for endocrine, 6 for rheumatological, 26 for hematological, and 46 for dermatological events. Because temporal completeness varied substantially and dates were not clinically adjudicated, these findings were considered exploratory.

## 4. Discussion

### 4.1. Principal Findings

This analysis identified heterogeneous autoimmune and immune-mediated reporting patterns among 4191 COVID-19 vaccine reports compared with 2817 traditional-vaccine reports. The strict subset comprised 311 unique reports contributing 316 report-category observations. Evidence was most coherent for hematological outcomes and, to a lesser extent, neurological outcomes; dermatological and endocrine findings were limited by sparse comparator data and weaker PT-level confirmation, rheumatological estimates were comparator- and suspect-role-sensitive, and strict cardiac events were absent.

These findings are pharmacovigilance signals rather than estimates of incidence, absolute risk, or causality. Interpretation therefore emphasizes coherence, precision, clustering, and stability across sensitivity analyses; detailed ROR values are presented in the Results and tables [4,10,11,12].

### 4.2. Interpretation of Category- and PT-Level Signals

Category- and PT-level results were most concordant for hematological and neurological outcomes. The hematological pattern was supported by immune thrombocytopenia and by acquired hemophilia before clustering was considered; the neurological pattern was supported by myasthenia gravis, optic neuritis, and multiple sclerosis. Acquired hemophilia nevertheless ranked highest largely because the comparator contained one event, and cluster collapse moved its estimate across the null (ROR 2.70, 95% CI 0.30–24.18). Immune thrombocytopenia therefore provided the stronger independent PT-level support for the hematological category.

The exact reanalysis retained the same five finite adjusted-positive PTs. It additionally identified Miller Fisher syndrome and acute disseminated encephalomyelitis as adjusted zero-cell exact imbalances in opposite directions, and juvenile idiopathic arthritis as an adjusted-supported lower finite finding. These changes did not produce finite ROR/PRR estimates for zero-cell tables or alter the classification of acquired hemophilia, myasthenia gravis, and optic neuritis as sparse/imprecise; the Yates sensitivity analysis retained the same five finite positive PTs (Table S20).

By contrast, dermatological and endocrine categories had high point estimates but sparse comparator counts and no adjusted-positive finite PT-level term. Their category-level findings should therefore be considered preliminary and aggregation-sensitive. Elevated finite estimates for systemic lupus erythematosus and autoimmune thyroiditis likewise did not satisfy the adjusted PT-level signal criterion.

Rheumatological reporting was lower than in the pooled active comparator under the primary PS + SS definition but reversed in the sparse PS-only analysis, indicating sensitivity to comparator composition and suspect-role classification. No strict cardiac autoimmune events were identified in either cohort. Together, these observations show why ranking by ROR magnitude cannot substitute for assessment of event counts, confidence-interval width, clustering, and consistency across sensitivity analyses.

### 4.3. Vaccine Platform-Specific Reporting Patterns

Platform-specific signals differed descriptively, but formal interaction supported heterogeneity only for endocrine events (mRNA-to-viral-vector ROR 0.22, 95% CI 0.08–0.64; *p* = 0.0052; BH-adjusted *p* = 0.0258), indicating higher relative reporting in the viral-vector stratum. No adjusted interaction was found for neurological, rheumatological, hematological, or dermatological events; therefore, stratum-specific significance alone was not considered evidence of platform differences.

These platform-specific findings should be interpreted cautiously. The mRNA stratum accounted for most COVID-19 vaccine reports, whereas the viral-vector stratum was much smaller, and no protein-subunit vaccine reports were present in the corrected platform-specific dataset. Consequently, some viral-vector estimates were based on sparse counts, and protein-subunit vaccine signals could not be assessed. In addition, FAERS does not contain administered-dose denominators, so platform-specific RORs cannot be used to infer comparative incidence or absolute risk across vaccine technologies. Rather, these results identify reporting patterns that may help prioritize follow-up analyses in denominator-linked data sources.

The product-specific findings were consistent with the platform analysis but also highlighted sparse-data limitations. Pfizer-BioNTech showed adjusted-supported dermatological, hematological, neurological, and endocrine findings, although the endocrine estimate remained sparse/imprecise. Moderna showed its clearest product-specific signal for hematological events, whereas AstraZeneca/Covishield showed significant endocrine and hematological disproportionality. Janssen and Novavax could not be meaningfully assessed because of very small or absent product-specific report counts. These product-level results should therefore be interpreted as descriptive pharmacovigilance patterns rather than evidence of causal differences between individual products.

### 4.4. Sex- and Age-Stratified Pattern

The strict autoimmune cohort showed female predominance overall, with 181 of 311 unique cases classified as female. Female predominance was most pronounced for endocrine events and was also observed for neurological and rheumatological categories. This pattern is consistent with the broader epidemiology of many autoimmune diseases, which often show sex-related differences in susceptibility, diagnosis, and reporting; however, FAERS data cannot distinguish biological susceptibility from differential healthcare use, diagnostic intensity, or reporting behavior [13,14,15].

Sex-stratified Fisher analyses showed adjusted-positive hematological and dermatological findings in both sexes and an additional neurological finding in females, but no sex interaction for these categories. Only rheumatological events showed an adjusted interaction (female-to-male ROR 0.30, 95% CI 0.14–0.64; BH *p* = 0.0070), with lower relative reporting in females and a non-significant estimate above unity in males. This exploratory result may reflect comparator composition or sex-related reporting behavior rather than biological effect modification.

Neurological reporting showed a significant age interaction. The <40-to-≥65 ROR was 12.53 (95% CI 3.63–43.29; BH-adjusted *p* = 0.00064), with a significant ordered trend (BH *p* = 0.00026). The ≥65-to-40–64 comparison was also significant (ROR 0.19, 95% CI 0.06–0.57; BH *p* = 0.0146), whereas the <40-to-40–64 comparison was not. These findings indicate an exploratory age-related difference in reporting disproportionality, not incidence or risk.

Hematological reporting showed a non-monotonic pattern. The nominally significant ≥65-to-40–64 interaction did not survive multiplicity correction (ROR 6.38, 95% CI 1.05–38.74; BH *p* = 0.147), and the trend test was non-significant. Dermatological reporting was adjusted-positive among individuals aged ≥65 years but remained sparse/imprecise, whereas endocrine estimates were limited by zero comparator cells. These stratum-specific classifications did not alter the formal age-interaction conclusions.

Age-stratified findings should not be interpreted as age-specific risk estimates. Older adults have different baseline disease rates, vaccine eligibility patterns, comorbidity profiles, healthcare utilization, and reporting probabilities than younger adults. Similarly, younger adults may differ in diagnostic workup and reporting of neurological symptoms. Denominator-linked cohort studies, self-controlled case series, and observed-versus-expected analyses are required to evaluate whether age-stratified reporting patterns correspond to differences in clinically confirmed risk [3,16,17].

### 4.5. Case-Definition Sensitivity and Misclassification

Case-definition sensitivity was driven mainly by inclusion of broad, non-specific PTs rather than by overlap between strict categories. Broader definitions increased comparator-event counts and changed the magnitude or direction of several estimates.

Neurological and hematological estimates attenuated under the broad definition, and the dermatological estimate no longer met the adjusted-positive criterion. Conversely, broad cardiac terms produced a signal despite the absence of strict autoimmune cardiac events. Broad PT sets can therefore either dilute a clinically specific pattern or generate a non-specific aggregate pattern, depending on their distribution across cohorts.

These findings support clinically specific PT dictionaries, transparent inclusion rules, PT-level review, and sensitivity analyses comparing strict and broad definitions [10,11,12].

### 4.6. Comparison with Previous Evidence

Previous VAERS, VigiBase, and EudraVigilance analyses have described autoimmune or serious adverse-event reporting across different vaccine products and clinical classes [6,7,8]. Direct numerical comparison is limited because databases differ in geographic coverage, reporting rules, product mix, duplicate handling, MedDRA coding, public attention, and comparator definitions.

The present active-comparator design, strict PT dictionary, subgroup interactions, PT-level ranking, and masking analyses emphasize clinical specificity, but they do not resolve the denominator and outcome-validation limitations of spontaneous reporting. Autoimmune hepatitis, considered in prior single-outcome studies [18,19], was outside the prespecified 48-term strict and 14-term broad-only dictionaries and therefore was not evaluated here.

Denominator-linked epidemiological studies provide the appropriate context for risk estimation. The Global Vaccine Data Network study used observed-versus-expected methods in 99 million vaccinated individuals, and controlled population-based studies have generally not shown broad increases across most autoimmune outcomes after mRNA vaccination [3,16]. FAERS therefore serves a complementary role: signals with adequate precision and clinical coherence can be prioritized for self-controlled, cohort, registry, or record-adjudication studies.

Interpretation must also account for prior or undiagnosed SARS-CoV-2 infection, pre-existing autoimmune disease, and concurrent triggers. Without infection history and clinical adjudication, reports listing a COVID-19 vaccine as a suspect product cannot distinguish these alternative explanations, particularly for neurological and systemic autoimmune outcomes [13,15,17,18,19,20].

### 4.7. Biological Plausibility of Autoimmune and Immune-Mediated Events

Proposed mechanisms include molecular mimicry, bystander immune activation, epitope spreading, interferon-pathway activation, and immune stimulation in susceptible individuals [13,14,15]. These mechanisms are biologically plausible but non-specific. Their interpretation requires concordant temporality, phenotype, rechallenge, alternative-cause assessment, laboratory confirmation, treatment response, and background incidence, details that are often incomplete in FAERS. Biological plausibility was therefore used only to prioritize hypotheses for clinical evaluation.

### 4.8. Implications for Post-Marketing Vaccine Safety Surveillance

Post-marketing surveillance should use transparent, clinically specific case definitions; pair category-level analyses with PT-level review; justify comparator composition; and evaluate platform, product, age, and sex only with formal interaction tests where counts permit. FAERS is suited to signal detection, whereas incidence estimation and hypothesis testing require active surveillance, linked claims or health records, vaccine registries, and clinical adjudication. Prioritization should integrate seriousness, event counts, precision, clustering, cross-database consistency, PT specificity, sensitivity-analysis stability, and feasibility of denominator-linked validation [1,2,3,4,5,10,11,12].

### 4.9. Strengths of the Study

This study has several strengths. It used a large FAERS background database covering 2021Q1 through 2023Q4 and applied harmonized COVID-19 vaccine product identification. It also used mutually exclusive platform classification to reduce double-counting and an active comparator group of traditional vaccine reports, which is more clinically relevant than comparing vaccine reports with all non-vaccine drug reports. The analysis included category-level, platform-specific, product-specific, sex-stratified, age-stratified, and PT-level components.

Another strength was the explicit evaluation of masking, dilution, and misclassification. By comparing strict and broad PT definitions, the analysis showed how broad definitions can attenuate true strict-category signals or create misleading broad-term findings. Sensitivity analyses using alternative comparator definitions, peak-period restriction, and primary-suspect-only restriction further clarified which findings were more robust and which were sensitive to analytic choices.

### 4.10. Limitations

This study has important limitations. FAERS is a spontaneous reporting system and is subject to underreporting, stimulated reporting, duplicate or follow-up submissions, missing information, and reporting bias. Reports may be influenced by media attention, regulatory communications, public awareness, clinician concern, product novelty, litigation, and differential reporting behavior. Therefore, the ROR and PRR estimates reported here reflect relative reporting disproportionality rather than incidence, absolute risk, or causal association [4,10,11,12].

The acquired-hemophilia audit highlighted a limitation of CASEID-based deduplication: one clinical episode may be submitted under different CASEIDs and MFR_NUMs by multiple manufacturers. Shared AUTH_NUMs and concordant profiles suggested cross-case duplication, but without a universal patient identifier or source narratives, the four clusters and probable cross-cohort link remain evidence-supported post hoc classifications rather than confirmed patient-level matches.

Seriousness coding differed between cohorts (89.6% of COVID-19 vaccine reports vs. 74.7% of comparator reports), potentially reflecting differences in populations, reporting pathways, or reporting intensity. In the serious-report-only analysis, neurological and endocrine estimates attenuated in magnitude but remained adjusted-supported under Fisher exact testing; hematological and dermatological findings and lower rheumatological reporting also persisted. Because restriction to serious reports may itself introduce selection bias, this analysis reduces but does not eliminate concern about differential reporting and should be viewed as a sensitivity analysis rather than a replacement for the primary analysis.

Suspect-role assignment was another source of sensitivity. PS-only restriction attenuated the neurological estimate, rendered endocrine, hematological, and dermatological estimates non-estimable, and reversed the rheumatological estimate from lower reporting to positive disproportionality. However, because this reversal was based on a single comparator event with a very wide confidence interval, it indicates instability under sparse role-restricted analysis rather than evidence of an opposite association.

Demographic composition and completeness differed between cohorts. Unknown age (35.3% vs. 13.8%) and sex (20.1% vs. 8.4%) were more frequent in comparator reports, resulting in greater exclusion from complete-case subgroup analyses. Comparator reports also included more individuals aged <40 years, whereas COVID-19 vaccine reports included more aged 40–64 and ≥65 years. These imbalances may affect unadjusted and subgroup estimates and could not be addressed through imputation; therefore, subgroup and interaction analyses were considered exploratory.

FAERS does not provide the number of administered vaccine doses or the number of exposed individuals. It also lacks complete information on diagnostic confirmation, laboratory testing, imaging, biopsy, electrophysiology, treatment, timing, rechallenge, recovery, and alternative causes. This limitation is particularly important for autoimmune conditions, which often require specialist confirmation and clinical adjudication. Misclassification may therefore remain possible even after applying a strict autoimmune PT dictionary. A suspect-product designation reflects reporter coding and does not independently establish that product administration preceded the coded event or that the product caused it.

Temporal data were incomplete and inconsistent. Only 132/311 reports (42.4%) had exact linked dates yielding a valid 0–365-day interval, while 68 had EVENT_DT preceding all matched vaccine START_DT values. Thus, the time-to-onset distribution represents a selected subset and cannot establish temporality or causality. Dose number, booster status, vaccination sequence, and heterologous schedules were also incomplete or inconsistently recorded; therefore, no dose-dependent or booster-specific conclusions are supported.

FAERS does not provide standardized post-vaccination follow-up; therefore, the 2021–2023 calendar window does not represent three years of observation for every report. Shorter follow-up for late-2023 vaccinations may have reduced detection of delayed or chronic conditions.

FAERS also lacks data on genetic susceptibility, including HLA status, latent infections (e.g., EBV or CMV), hormonal factors, psychosocial stress, and complete SARS-CoV-2 infection history, including PCR confirmation. These unmeasured factors limit attribution of reported autoimmune events to a single trigger. Residual confounding from prior or undiagnosed infection, pre-existing autoimmune disease, immunosuppressive therapy, comorbidities, healthcare use, and vaccine eligibility remains. Sparse counts and zero comparator cells limited several endocrine, dermatological, product-specific, and subgroup estimates. The heterogeneous comparator included 206 records excluded by exact-token audit: 147 non-vaccine substring matches, 47 unclassifiable unspecified-vaccine reports, 10 therapeutic/experimental terms, and two COVID-19 leakage records. Because the 2817-report cohort was prespecified, it remained primary, whereas the post hoc 2611-report product-classifiable cohort was used for sensitivity analysis. Primary findings were materially unchanged, but pooled estimates should be interpreted relative to the observed mixture of traditional vaccines rather than a homogeneous class.

### 4.11. Future Research Directions

Future studies should validate the main adjusted PT-level signals—acquired hemophilia, immune thrombocytopenia, myasthenia gravis, optic neuritis, and multiple sclerosis—in denominator-linked datasets. Priority designs include self-controlled case series, observed-versus-expected analyses, active-comparator cohort studies, and nested case–control studies using electronic health records, claims data, vaccine registries, and active surveillance systems. Such studies should incorporate age, sex, vaccine product, dose number, time since vaccination, prior SARS-CoV-2 infection, comorbidities, autoimmune disease history, and immunosuppressive therapy. For acquired hemophilia, validation should include source-level linkage and clinical adjudication to distinguish independent patients from parallel manufacturer submissions.

Additional work should evaluate the dermatological and endocrine category-level findings in larger denominator-linked epidemiological studies with validated outcomes and clinical adjudication, because these findings relied on only two comparator events and lacked adjusted-positive finite PT-level confirmation. Future pharmacovigilance studies should also report both strict and broad case definitions, provide PT-level tables, and evaluate masking and dilution explicitly. Standardized reporting according to contemporary disproportionality-analysis guidance would improve comparability across FAERS, VAERS, EudraVigilance, VigiBase, and other international pharmacovigilance systems [10,11,12].

## 5. Conclusions

In this FAERS-based active-comparator disproportionality analysis, selected autoimmune and immune-mediated MedDRA Preferred Terms were reported disproportionately among reports listing COVID-19 vaccines compared with reports listing traditional vaccines. The hematological positive signal had the most stable robustness profile. Lower rheumatological reporting was supported in the primary and several sensitivity analyses but reversed in the sparse PS-only analysis, indicating sensitivity to suspect-product classification. The neurological finding was intermediate, and dermatological and endocrine category-level findings were sparse/imprecise and require validation. Because FAERS lacks exposure denominators and clinical adjudication, these findings should be considered hypothesis-generating reporting signals rather than evidence of incidence, absolute risk, or causality.

Fisher exact reanalysis retained the same five finite adjusted-positive PTs: immune thrombocytopenia was more stable, multiple sclerosis intermediate, and acquired hemophilia, myasthenia gravis, and optic neuritis sparse/imprecise. Miller Fisher syndrome showed an adjusted higher-reporting exact imbalance with a zero-comparator cell, so ROR/PRR remained non-estimable, and it was excluded from finite rankings. Dermatological and endocrine category signals lacked finite PT-level confirmation, while cluster collapse attenuated acquired hemophilia to a non-significant, highly imprecise estimate (Fisher *p* = 0.654), arguing against a robust, broadly distributed signal.

The masking and sensitivity analyses demonstrated that broad case definitions can dilute strict autoimmune signals or introduce non-specific signals, particularly for dermatological, neurological, hematological, and cardiac categories. Hematological events were the most consistent finite signal across sensitivity scenarios, while cardiac findings appeared only under broad definitions and should not be interpreted as strict autoimmune cardiac signals.

These findings support the value of refined pharmacovigilance approaches for autoimmune signal detection, including strict autoimmune definitions, active-comparator designs, platform-specific analyses, demographic stratification, PT-level ranking, and sensitivity analyses. Because FAERS is a spontaneous reporting system, the results are hypothesis-generating and should not be interpreted as evidence of causality, incidence, or absolute risk. Future vaccine safety surveillance should integrate spontaneous reporting signals with active surveillance, denominator-linked epidemiological studies, and clinical adjudication. Future presentations of ranked disproportionality findings should report event counts, interval precision, and robustness tier alongside effect magnitude.

## Figures and Tables

**Table 1:** Distribution of COVID-19 Vaccine Reports by Product and Platform.

**Panel A. Mutually exclusive COVID-19 vaccine product distribution**					
**Vaccine product/brand group**	**Platform/product group**	**Total COVID-19 PS + SS reports, *n* (%)**	**Serious COVID-19 PS + SS reports, *n* (%)**	**Strict autoimmune reports, *n* (%)**	**Serious strict autoimmune reports, *n* (%)**
Pfizer-BioNTech	mRNA	3431 (81.9)	3019 (88.0)	246 (79.1)	246 (100.0)
Moderna	mRNA	291 (6.9)	274 (94.2)	21 (6.8)	20 (95.2)
AstraZeneca	Viral vector	367 (8.8)	364 (99.2)	32 (10.3)	32 (100.0)
Janssen	Viral vector	17 (0.4)	17 (100.0)	0 (0.0)	N/E
Multiple COVID-19 vaccine brands	Multiple/overlapping products	85 (2.0)	83 (97.6)	12 (3.9)	12 (100.0)
Total	—	4191 (100.0)	3757 (89.6)	311 (100.0)	310 (99.7)
**Panel B. Corrected mutually exclusive vaccine-platform distribution**					
**Vaccine platform**	**Included product groups**		**Total COVID-19 PS + SS reports, *n* (%)**	**Strict autoimmune report-category observations, *n* (%)**	**Category composition of strict autoimmune observations**
mRNA	COMIRNATY/Pfizer-BioNTech; SPIKEVAX/Moderna; corrected platform-assigned overlapping reports		3807 (90.8)	283 (89.6)	Neurological 110; rheumatological 80; hematological 53; dermatological 29; endocrine 11; cardiac 0
Viral vector	VAXZEVRIA/AstraZeneca/Covishield; AD26.COV2.S/Janssen		384 (9.2)	33 (10.4)	Neurological 5; rheumatological 12; hematological 9; dermatological 2; endocrine 5; cardiac 0
Protein subunit	NUVAXOVID/Novavax		0 (0.0)	0 (0.0)	Not estimable
Total	—		4191 (100.0)	316 (100.0)	Neurological 115; rheumatological 92; hematological 62; dermatological 31; endocrine 16; cardiac 0
**Panel C. Demographic and reporting-characteristic comparison of the complete exposure cohorts**					
**Characteristic**		**COVID-19 vaccine cohort (*N* = 4191)**	**Traditional-vaccine comparator cohort (*N* = 2817)**		**Absolute difference, percentage points**
Age, median (IQR), years		61 (46–73)	55 (11–70)		N/A
Age available		3613 (86.2)	1823 (64.7)		21.5
Age <40 years		617 (14.7)	682 (24.2)		−9.5
Age 40–64 years		1465 (35.0)	449 (15.9)		19
Age >= 65 years		1531 (36.5)	692 (24.6)		12
Age unknown		578 (13.8)	994 (35.3)		−21.5
Sex available		3838 (91.6)	2250 (79.9)		11.7
Female		2370 (56.6)	1374 (48.8)		7.8
Male		1468 (35.0)	876 (31.1)		3.9
Sex unknown		353 (8.4)	567 (20.1)		−11.7
Serious report		3757 (89.6)	2105 (74.7)		14.9

Note. PS + SS, primary suspect plus secondary suspect; N/E, not estimable. Product rows are mutually exclusive. Platform denominators were corrected to avoid double-counting reports containing more than one COVID-19 vaccine platform term. Percentages in Panel A are calculated using 4191 COVID-19 PS + SS reports and 311 strict autoimmune reports. Percentages in Panel B are calculated using 4191 COVID-19 PS + SS reports and 316 strict autoimmune report-category observations. Seriousness was defined using FAERS OUTC codes; individual OUTC codes were not mutually exclusive, whereas the binary serious/non-serious classification was mutually exclusive at the report level. Panel C percentages use the full cohort denominators. Median and IQR are calculated among reports with known age. Unknown age and sex were retained in the overall cohorts but excluded from the corresponding stratified analyses; no imputation was performed. Absolute differences are COVID minus comparator percentage points.

**Table 2:** Category-Level Disproportionality Signals for Autoimmune Events Among FAERS Reports Listing COVID-19 Vaccines as Suspect Products.

Autoimmune Category	A: COVID + Event	B: COVID + No Event	C: Comparator + Event	D: Comparator + No Event	ROR (95% CI)	PRR (95% CI)	Fisher–BH *p*	Interpretation
Neurological	115	4076	48	2769	1.63 (1.16–2.29)	1.61 (1.15–2.25)	0.0057	Positive signal; intermediate robustness
Endocrine	16	4175	2	2815	5.39 (1.24–23.48)	5.38 (1.24–23.37)	0.0138	Positive signal; sparse/imprecise
Rheumatological	92	4099	102	2715	0.60 (0.45–0.80)	0.61 (0.46–0.80)	7.64 × 10^−4^	Lower reporting; more stable; PS-only reversal
Hematological	62	4129	9	2808	4.68 (2.32–9.44)	4.63 (2.31–9.30)	2.73 × 10^−6^	Positive signal; more stable
Dermatological	31	4160	2	2815	10.49 (2.51–43.86)	10.42 (2.50–43.50)	4.42 × 10^−5^	Positive signal; sparse/imprecise
Cardiac	0	4191	0	2817	N/E	N/E	N/A	Not estimable

Note: A, COVID-19 vaccine reports with the event; B, COVID-19 vaccine reports without the event; C, traditional vaccine comparator reports with the event; D, traditional vaccine comparator reports without the event; ROR, reporting odds ratio; PRR, proportional reporting ratio; CI, confidence interval; N/E, not estimable. Raw *p* values were obtained using two-sided Fisher exact tests, and adjusted *p* values were Benjamini–Hochberg corrected across the five non-degenerate primary category tables. The cardiac row had A = C = 0 and was not tested. BH: Benjamini–Hochberg; PS: primary suspect.

**Table 3:** Vaccine Platform-Specific ROR and PRR Estimates for Autoimmune Event Categories.

Platform	Category	Platform Events, *n*/*N* (%)	Comparator Events, *n*/*N* (%)	ROR (95% CI)	PRR (95% CI)	Pooled BH *p*	Within-Platform BH *p*	Interpretation
mRNA	Neurological	110/3807 (2.89)	48/2817 (1.70)	1.72 (1.22–2.42)	1.70 (1.21–2.37)	0.00314	0.00236	Positive signal
mRNA	Endocrine	11/3807 (0.29)	2/2817 (0.07)	4.08 (0.90–18.42)	4.07 (0.90–18.35)	0.075	0.0525	No positive signal
mRNA	Rheumatological	80/3807 (2.10)	102/2817 (3.62)	0.57 (0.42–0.77)	0.58 (0.43–0.77)	0.00061	0.000407	Lower vs. comparator
mRNA	Hematological	53/3807 (1.39)	9/2817 (0.32)	4.40 (2.17–8.94)	4.36 (2.15–8.82)	3.36 × 10^−5^	1.68 × 10^−5^	Positive signal
mRNA	Dermatological	29/3807 (0.76)	2/2817 (0.07)	10.80 (2.58–45.32)	10.73 (2.56–44.93)	5.98 × 10^−5^	2.99 × 10^−5^	Positive signal
mRNA	Cardiac	0/3807 (0.00)	0/2817 (0.00)	N/E	N/E	N/E	N/E	Not estimable
Viral vector	Neurological	5/384 (1.30)	48/2817 (1.70)	0.76 (0.30–1.92)	0.76 (0.31–1.91)	0.749	0.769	No positive signal
Viral vector	Endocrine	5/384 (1.30)	2/2817 (0.07)	18.57 (3.59–96.05)	18.34 (3.57–94.20)	0.000828	0.00104	Positive signal
Viral vector	Rheumatological	12/384 (3.12)	102/2817 (3.62)	0.86 (0.47–1.58)	0.86 (0.48–1.55)	0.769	0.769	No positive signal
Viral vector	Hematological	9/384 (2.34)	9/2817 (0.32)	7.49 (2.95–18.98)	7.34 (2.93–18.37)	0.000282	0.000423	Positive signal
Viral vector	Dermatological	2/384 (0.52)	2/2817 (0.07)	7.37 (1.03–52.47)	7.34 (1.04–51.93)	0.0913	0.122	No positive signal
Viral vector	Cardiac	0/384 (0.00)	0/2817 (0.00)	N/E	N/E	N/E	N/E	Not estimable

Note. Platform denominators were mutually exclusive. Raw *p* values were obtained using two-sided Fisher exact tests. The pooled Fisher–Benjamini–Hochberg column was corrected across the 10 non-degenerate platform-category tests; the within-platform column was corrected separately across five non-degenerate tests per platform. No protein-subunit reports were present. ROR, reporting odds ratio; PRR, proportional reporting ratio; CI, confidence interval; BH, Benjamini–Hochberg; PS + SS, primary suspect plus secondary suspect; N/E, not estimable. Formal mRNA-versus-viral-vector interaction testing used the ratio of RORs and retained the prespecified Wald procedure. Only the endocrine interaction remained supported after correction (ROR 0.22, 95% CI 0.08–0.64; *p* = 0.0052; BH *p* = 0.0258); all other platform-interaction BH *p* values were ≥0.193 (Table S17).

**Table 4:** Sex-Stratified ROR and PRR Estimates for Autoimmune Event Categories.

Stratum	Autoimmune Category	A: COVID + Event	C: Comparator + Event	COVID Denominator	Comparator Denominator	ROR (95% CI)	PRR (95% CI)	Fisher–BH *p*	Interpretation
Female	Neurological	72	24	2370	1374	1.76 (1.10–2.81)	1.74 (1.10–2.75)	0.0357	Positive signal
Female	Endocrine	14	2	2370	1374	4.08 (0.93–17.96)	4.06 (0.92–17.83)	0.0932	Elevated estimate; not adjusted-positive
Female	Rheumatological	55	71	2370	1374	0.44 (0.30–0.62)	0.45 (0.32–0.63)	5.26 × 10^−5^	Lower reporting vs. comparator
Female	Hematological	29	3	2370	1374	5.66 (1.72–18.62)	5.60 (1.71–18.36)	0.00353	Positive signal
Female	Dermatological	14	1	2370	1374	8.16 (1.07–62.11)	8.12 (1.07–61.66)	0.0357	Positive signal; sparse/imprecise
Female	Cardiac	0	0	2370	1374	N/E	N/E	N/E	Not estimable
Male	Neurological	32	17	1468	876	1.13 (0.62–2.04)	1.12 (0.63–2.01)	0.852	No positive signal
Male	Endocrine	1	0	1468	876	N/E	N/E	1.000	No adjusted exact imbalance; ROR/PRR N/E
Male	Rheumatological	31	13	1468	876	1.43 (0.75–2.75)	1.42 (0.75–2.70)	0.432	No positive signal
Male	Hematological	30	4	1468	876	4.55 (1.60–12.95)	4.48 (1.58–12.66)	0.00383	Positive signal
Male	Dermatological	13	1	1468	876	7.82 (1.02–59.87)	7.76 (1.02–59.20)	0.0387	Positive signal; sparse/imprecise
Male	Cardiac	0	0	1468	876	N/E	N/E	N/E	Not estimable

Note: A, COVID-19 vaccine reports with the event; C, traditional vaccine comparator reports with the event; ROR, reporting odds ratio; PRR, proportional reporting ratio; CI, confidence interval; N/E, not estimable. Reports with unknown sex were retained in the audit dataset but excluded from the primary female-versus-male comparison. Raw *p* values were obtained using two-sided Fisher exact tests, and Benjamini–Hochberg adjustment was applied across the 10 non-degenerate sex-category tables. For the male endocrine row (C = 0), ROR and PRR were N/E, and the Fisher exact result was not adjusted-supported. Cardiac rows (A = C = 0) were not tested. Formal female-to-male interaction tests retained the prespecified Wald procedure; only rheumatological events remained supported after correction (ROR 0.30, 95% CI 0.14–0.64; *p* = 0.0018; BH *p* = 0.0070).

**Table 5:** Age-Stratified ROR and PRR Estimates for Autoimmune Event Categories.

Stratum	Autoimmune Category	A: COVID + Event	C: Comparator + Event	COVID Denominator	Comparator Denominator	ROR (95% CI)	PRR (95% CI)	Fisher–BH *p*	Interpretation
<40	Neurological	23	8	617	682	3.26 (1.45–7.35)	3.18 (1.43–7.05)	0.0108	Positive signal
<40	Endocrine	9	0	617	682	N/E	N/E	0.0056	Adjusted exact higher-reporting imbalance; ROR/PRR N/E
<40	Rheumatological	3	7	617	682	0.47 (0.12–1.83)	0.47 (0.12–1.82)	0.487	No positive signal
<40	Hematological	7	1	617	682	7.81 (0.96–63.70)	7.74 (0.95–62.71)	0.0539	Elevated estimate; not adjusted-positive
<40	Dermatological	0	0	617	682	N/E	N/E	N/A	Not estimable
<40	Cardiac	0	0	617	682	N/E	N/E	N/A	Not estimable
40–64	Neurological	70	16	1465	449	1.36 (0.78–2.36)	1.34 (0.79–2.28)	0.468	Elevated estimate; not adjusted-positive
40–64	Endocrine	5	1	1465	449	1.53 (0.18–13.17)	1.53 (0.18–13.08)	1.000	Elevated estimate; not adjusted-positive
40–64	Rheumatological	38	32	1465	449	0.35 (0.21–0.56)	0.36 (0.23–0.58)	5.00 × 10^−4^	Lower reporting vs. comparator
40–64	Hematological	16	4	1465	449	1.23 (0.41–3.69)	1.23 (0.41–3.65)	1.000	Elevated estimate; not adjusted-positive
40–64	Dermatological	9	1	1465	449	2.77 (0.35–21.92)	2.76 (0.35–21.71)	0.596	Elevated estimate; not adjusted-positive
40–64	Cardiac	0	0	1465	449	N/E	N/E	N/A	Not estimable
≥65	Neurological	7	12	1531	692	0.26 (0.10–0.66)	0.26 (0.10–0.67)	0.0130	Lower reporting vs. comparator
≥65	Endocrine	1	0	1531	692	N/E	N/E	1.000	No adjusted exact imbalance; ROR/PRR N/E
≥65	Rheumatological	32	26	1531	692	0.55 (0.32–0.92)	0.56 (0.33–0.93)	0.0539	Lower estimate; not adjusted-significant
≥65	Hematological	34	2	1531	692	7.84 (1.88–32.71)	7.68 (1.85–31.89)	0.0026	Positive signal
≥65	Dermatological	17	1	1531	692	7.76 (1.03–58.42)	7.68 (1.02–57.62)	0.0441	Positive signal; sparse/imprecise
≥65	Cardiac	0	0	1531	692	N/E	N/E	N/A	Not estimable

Note. A, COVID-19 vaccine reports with the event; C, traditional vaccine comparator reports with the event; ROR, reporting odds ratio; PRR, proportional reporting ratio; CI, confidence interval; N/E, not estimable. Age strata were predefined as <40, 40–64, and ≥65 years; reports with unknown age were retained in the audit dataset but excluded from the primary age-stratified comparison. Fisher exact *p* values were adjusted using Benjamini–Hochberg correction across 14 computable age-stratified tests; rows with A = C = 0 were not tested.

**Table 6:** Top 10 Autoimmune Preferred Terms Ranked by Finite Reporting Odds Ratio. Robustness tier is shown in the interpretation column.

Rank	Preferred Term	Category	A: COVID Event	C: Comparator Event	ROR (95% CI)	PRR (95% CI)	Fisher–BH *p*	Interpretation
1	Acquired hemophilia	Hematological	20	1	13.50 (1.81–100.67)	13.44 (1.81–100.11)	0.0032	Positive signal; sparse/imprecise, cluster-sensitive: 20 reports formed four potential episodes; cluster-collapsed ROR 2.70 (95% CI 0.30–24.18)
2	Myasthenia gravis	Neurological	19	2	6.41 (1.49–27.54)	6.39 (1.49–27.39)	0.0117	Positive signal; sparse/imprecise
3	Optic neuritis	Neurological	22	3	4.95 (1.48–16.55)	4.93 (1.48–16.45)	0.0117	Positive signal; sparse/imprecise
4	Immune thrombocytopenia	Hematological	37	8	3.13 (1.45–6.73)	3.11 (1.45–6.67)	0.0088	Positive signal; more stable
5	Multiple sclerosis	Neurological	55	12	3.11 (1.66–5.81)	3.08 (1.65–5.74)	0.0018	Positive signal; intermediate
6	Autoimmune thyroiditis	Endocrine	5	2	1.68 (0.33–8.67)	1.68 (0.33–8.66)	0.7364	No adjusted positive signal; sparse/imprecise
7	Systemic lupus erythematosus	Rheumatological	42	19	1.49 (0.87–2.57)	1.49 (0.87–2.55)	0.2688	No adjusted positive signal; intermediate
8	Ankylosing spondylitis	Rheumatological	3	5	0.40 (0.10–1.69)	0.40 (0.10–1.69)	0.3791	No adjusted positive signal; sparse/imprecise
9	Polymyalgia rheumatica	Rheumatological	5	9	0.37 (0.12–1.11)	0.37 (0.13–1.11)	0.1476	No adjusted positive signal; more stable estimate
10	Rheumatoid arthritis	Rheumatological	38	71	0.35 (0.24–0.53)	0.36 (0.24–0.53)	4.64 × 10^−6^	Lower reporting; more stable estimate

Note. Ranking was based on finite ROR magnitude and does not represent evidentiary strength. Preferred Terms with zero comparator events were not ranked because finite ROR and PRR estimates could not be calculated from the observed counts. Fisher exact testing nevertheless evaluated non-degenerate zero-cell tables; adjusted-supported zero-cell results were reported as exact imbalances rather than finite signals. Sparse/imprecise indicates A or C < 5 or a 95% CI fold-width > 10; more stable additionally requires parent-category result consistency in at least three of five sensitivity analyses; remaining finite estimates are intermediate. The acquired-hemophilia rank uses the prespecified report-level table; Table S18 presents the post hoc independence audit and cluster-collapsed sensitivity analysis, and Table S20 compares Fisher and Yates inference.

## Data Availability

Publicly available FAERS quarterly data were used in this study and are available from the U.S. Food and Drug Administration FAERS database. The analytic code and derived dictionaries are available from the corresponding authors upon reasonable request.

## Supplementary Materials

The following supporting information can be downloaded at: https://www.mdpi.com/article/10.3390/ijerph23081088/s1, Figure S1: FAERS Cohort Construction and Autoimmune Category Counting; Table S1: Strict and Broad-Only MedDRA Preferred Terms Used for Autoimmune Event Classification; Table S2: Cohort-Level Characteristics of FAERS Reports Included in the Analysis; Table S3: Characteristics of Strict Autoimmune Observations Among Reports Listing a COVID-19 Vaccine as a Suspect Product, by Category; Table S4: Seriousness Distribution Among Strict Autoimmune Categories; Table S5: Distribution of Seriousness Outcomes by Cohort; Table S6: Product-Specific ROR and PRR Estimates for Autoimmune Event Categories Among Reports Listing COVID-19 Vaccines as Suspect Products; Table S7: Age-Interaction Analysis Based on ROR; Table S8: Preferred Term-Level Disproportionality Analysis of Autoimmune Events; Table S9: Concordance Between Category-Level and PT-Level Signal Detection; Table S10: Discordance Between Category-Level and PT-Level Signal Detection; Table S11: Sensitivity Analyses of Autoimmune Safety Signals; Table S12: Prespecified Product Terms and Rules Used for Vaccine Cohort Identification; Table S13: Composition of the protocol-screened traditional non-COVID vaccine comparator cohort by harmonized vaccine class and product; Table S14: Strict autoimmune category-level disproportionality estimates using pooled and major traditional-vaccine comparator definitions; Table S15: Serious-Report-Only Sensitivity Analysis of Category-Level Autoimmune Disproportionality; Table S16: Evidence Robustness Classification for Category-Level and Top-Ranked PT Findings; Table S17: Formal Interaction Tests for Vaccine Platform, Sex, and Age; Table S18: Independence, Reporting Concentration, and Potential Clinical-Episode Clustering Audit for Acquired Hemophilia; Table S19: Completeness and Descriptive Time to Onset Among Strict-Autoimmune Reports Listing a COVID-19 Vaccine as a Suspect Product; Table S20: Comparison of Fisher Exact and Yates-Corrected Inference for the Primary Category-Level and Strict PT-Level Families.
