# Supplementary material for: Autoimmune Reporting Signals in FAERS Reports Listing COVID-19 Vaccines as Suspect Products: An Active-Comparator Disproportionality Analysis
Source: Int J Environ Res Public Health. 2026 Aug 21;23(8):1088. doi: 10.3390/ijerph23081088 (PMC13513629; doi:10.3390/ijerph23081088)
Supplement: Supplementary file 1 [file ijerph-23-01088-s001.zip › ijerph-4410111-supplementary.pdf]

Table S1. Strict and Broad-Only MedDRA Preferred Terms Used for Autoimmune and Immune-Mediated Event Classification.

Category	MedDRA Preferred Term	Included in Strict Definition
Neurological	Acute disseminated encephalomyelitis	Yes
Neurological	Autoimmune encephalitis	Yes
Neurological	Autoimmune neuropathy	Yes
Neurological	Guillain-Barre syndrome	Yes
Neurological	Miller Fisher syndrome	Yes
Neurological	Multiple sclerosis	Yes
Neurological	Myasthenia gravis	Yes
Neurological	Neuromyelitis optica spectrum disorder	Yes
Neurological	Optic neuritis	Yes
Neurological	Transverse myelitis	Yes
Neurological	Demyelination	No
Neurological	Encephalitis noninfective	No
Neurological	Peripheral neuropathy	No
Endocrine	Addison's disease	Yes
Endocrine	Autoimmune adrenalitis	Yes
Endocrine	Autoimmune hypophysitis	Yes
Endocrine	Autoimmune insulin syndrome	Yes
Endocrine	Autoimmune thyroiditis	Yes
Endocrine	Basedow's disease	Yes
Endocrine	Graves' disease	Yes
Endocrine	Hashimoto's thyroiditis	Yes
Endocrine	Polyglandular autoimmune syndrome type I	Yes
Endocrine	Polyglandular autoimmune syndrome type II	Yes
Endocrine	Type 1 diabetes mellitus	Yes
Rheumatological	Ankylosing spondylitis	Yes
Rheumatological	Behcet's syndrome	Yes
Rheumatological	Dermatomyositis	Yes
Rheumatological	Juvenile idiopathic arthritis	Yes
Rheumatological	Polymyalgia rheumatica	Yes
Rheumatological	Polymyositis	Yes
Rheumatological	Reactive arthritis	Yes
Rheumatological	Rheumatoid arthritis	Yes
Rheumatological	Sjogren's syndrome	Yes
Rheumatological	Systemic lupus erythematosus	Yes

Rheumatological	Systemic sclerosis	Yes
Rheumatological	Psoriatic arthropathy	No
Rheumatological	Vasculitis	No
Hematological	Acquired haemophilia	Yes
Hematological	Autoimmune haemolytic anaemia	Yes
Hematological	Autoimmune neutropenia	Yes
Hematological	Evans syndrome	Yes
Hematological	Immune thrombocytopenia	Yes
Hematological	Pure red cell aplasia	Yes
Hematological	Aplastic anaemia	No
Hematological	Haemolytic anaemia	No
Hematological	Neutropenia	No
Hematological	Thrombocytopenia	No
Dermatological	Alopecia areata	Yes
Dermatological	Autoimmune dermatitis	Yes
Dermatological	Bullous pemphigoid	Yes
Dermatological	Cutaneous lupus erythematosus	Yes
Dermatological	Lichen planus	Yes
Dermatological	Morphea	Yes
Dermatological	Pemphigus	Yes
Dermatological	Vitiligo	Yes
Dermatological	Psoriasis	No
Cardiac	Autoimmune myocarditis	Yes
Cardiac	Autoimmune pericarditis	Yes
Cardiac	Myocarditis	No
Cardiac	Myopericarditis	No
Cardiac	Pericarditis	No
Cardiac	Pleuropericarditis	No

Note: PT, Preferred Term. This table contains 48 strict PTs used for the primary autoimmune definition and 14 broad-only PTs reserved for sensitivity analyses. Broad-only terms were not included in the primary strict autoimmune definition. Basedow's disease and Graves' disease were retained as separate PT labels in the source REAC data. Report-level review identified no CASEID containing both terms, and within-category counting allowed each report to contribute no more than one endocrine observation.

Table S2. Cohort-Level Characteristics of FAERS Reports Included in the Analysis. Panel A. Cohort-level characteristics

Characteristic	COVID-19 Vaccine PS+SS cohort	Traditional Vaccine Comparator PS+SS Cohort	Strict Autoimmune COVID-19 Cohort
Study period	2021Q1–2023Q4	2021Q1–2023Q4	2021Q1–2023Q4
Reports/cases, n	4191	2817	311
Report-category observations, n	—	—	316
Primary-suspect-only subset, n	735	485	—
Age available, n (%)	3613 (86.2)	1,823 (64.7)	266 (85.5)
Age, median (IQR), years	61.0 (46.0–73.0)	55.0 (11.0–70.0)	59.0 (44.2–68.0)
Female sex, n (%)	2370 (56.5)	1374 (48.8)	181 (58.2)
Male sex, n (%)	1468 (35.0)	876 (31.1)	105 (33.8)
Unknown sex, n (%)	353 (8.4)	567 (20.1)	25 (8.0)
Serious reports/cases, n (%)	3757 (89.6)	2105 (74.7)	310 (99.7)
Non-serious/no serious OUTC coded, n (%)	434 (10.4)	712 (25.3)	1 (0.3)

Table S3. Characteristics of Strict Autoimmune Observations Among Reports Listing a COVID-19 Vaccine as a Suspect Product, by Category

Autoimmune Category	Report-Category Observations, n	Age, Median (IQR), Years	Female, n (%)	Male, n (%)	Unknown sex, n (%)	Serious, n (%)	Non-Serious/No Serious OUTC Coded, n (%)
Neurological	115	48.5 (40.0–56.2)	72 (62.6)	32 (27.8)	11 (9.6)	115 (100.0)	0 (0.0)
Endocrine	16	30.0 (30.0–53.0)	14 (87.5)	1 (6.2)	1 (6.2)	16 (100.0)	0 (0.0)
Rheumatological	92	63.0 (53.0–73.0)	55 (59.8)	31 (33.7)	6 (6.5)	91 (98.9)	1 (1.1)
Hematological	62	67.0 (61.0–81.0)	29 (46.8)	30 (48.4)	3 (4.8)	62 (100.0)	0 (0.0)
Dermatological	31	66.0 (64.0–77.0)	14 (45.2)	13 (41.9)	4 (12.9)	31 (100.0)	0 (0.0)
Cardiac	0	—	0	0	0	—	—

Note: FAERS, FDA Adverse Event Reporting System; PS, primary suspect; SS, secondary suspect; OUTC, outcome code; IQR, interquartile range. The strict-autoimmune subset contains 311 unique reports listing a COVID-19 vaccine as a PS or SS suspect product and 316 report-category observations because some reports contributed to more than one autoimmune category. Percentages are calculated using the column denominator in Panel A and the category denominator in Panel B. Cardiac strict autoimmune events were not observed in the strict cohort. Source: FAERS protocol reanalysis 20260528 and serious/non-serious outcome summary files.

Table S4. Seriousness Distribution Among Strict Autoimmune Categories.

Autoimmune Category	Total Observations	Serious	Non-Serious / No Serious OUTC Coded	Serious, %
Dermatological	31	31	0	100.0
Endocrine	16	16	0	100.0
Hematological	62	62	0	100.0
Neurological	115	115	0	100.0
Rheumatological	92	91	1	98.9

Table S5. Distribution of Seriousness Outcomes by Cohort.

Cohort	Seriousness Outcome	OUTC Code	Number of Reports	Percentage	Denominator
COVID PS+SS	Death	DE	441	10.5	4,191
COVID PS+SS	Life-threatening	LT	337	8.0	4,191
COVID PS+SS	Hospitalization	HO	1,751	41.8	4,191
COVID PS+SS	Disability	DS	177	4.2	4,191
COVID PS+SS	Congenital anomaly	CA	28	0.7	4,191
COVID PS+SS	Required intervention	RI	0	0.0	4,191
COVID PS+SS	Other serious outcome	OT	2,467	58.9	4,191
COVID PS+SS	Non-serious / no serious OUTC recorded	—	434	10.4	4,191
COVID strict autoimmune	Death	DE	2	0.6	311
COVID strict autoimmune	Life-threatening	LT	22	7.1	311
COVID strict autoimmune	Hospitalization	HO	166	53.4	311
COVID strict autoimmune	Disability	DS	16	5.1	311
COVID strict autoimmune	Congenital anomaly	CA	0	0.0	311
COVID strict autoimmune	Required intervention	RI	0	0.0	311
COVID strict autoimmune	Other serious outcome	OT	256	82.3	311
COVID strict autoimmune	Non-serious / no serious OUTC recorded	—	1	0.3	311
Traditional comparator PS+SS	Death	DE	140	5.0	2,817
Traditional comparator PS+SS	Life-threatening	LT	93	3.3	2,817
Traditional comparator PS+SS	Hospitalization	HO	833	29.6	2,817
Traditional comparator PS+SS	Disability	DS	152	5.4	2,817
Traditional comparator PS+SS	Congenital anomaly	CA	76	2.7	2,817
Traditional comparator PS+SS	Required intervention	RI	3	0.1	2,817

Traditional comparator PS+SS	Other serious outcome	OT	1,742	61.8	2,817
Traditional comparator PS+SS	Non-serious / no serious OUTC recorded	—	712	25.3	2,817

Note: Individual FAERS OUTC codes are not mutually exclusive; one report may include multiple serious outcome codes. Therefore, percentages for individual outcome categories may sum to more than 100%. The “Non-serious / no serious OUTC recorded” row represents the mutually exclusive binary non-serious category.

Table S6. Product-Specific ROR and PRR Estimates for Autoimmune Event Categories Among Reports Listing COVID-19 Vaccines as Suspect Products.

Product	Category	A Product Event	C Comparator Event	N Product	N Comparator	ROR (95% CI)	PRR (95% CI)	Fisher p	Fisher–BH p
Pfizer-BioNTech	Neurological	98	48	3431	2817	1.70 (1.20–2.40)	1.68 (1.19–2.36)	0.0031	0.00387
Pfizer-BioNTech	Endocrine	11	2	3431	2817	4.53 (1.00–20.44)	4.52 (1.00–20.36)	0.047	0.047
Pfizer-BioNTech	Rheumatological	75	102	3431	2817	0.59 (0.44–0.80)	0.60 (0.45–0.81)	0.000725	0.00124
Pfizer-BioNTech	Hematological	36	9	3431	2817	3.31 (1.59–6.88)	3.28 (1.58–6.81)	0.000743	0.00124
Pfizer-BioNTech	Dermatological	29	2	3431	2817	12.00 (2.86–50.33)	11.91 (2.84–49.85)	4.16×10^{-6}	2.08×10^{-5}
Pfizer-BioNTech	Cardiac	0	0	3431	2817	N/E	N/E	N/E	N/E
Moderna	Neurological	7	48	291	2817	1.42 (0.64–3.17)	1.41 (0.64–3.09)	0.351	0.585
Moderna	Endocrine	0	2	291	2817	N/E	N/E	1.000	1.000
Moderna	Rheumatological	2	102	291	2817	0.18 (0.05–0.75)	0.19 (0.05–0.77)	0.00519	0.013
Moderna	Hematological	13	9	291	2817	14.59 (6.18–34.44)	13.98 (6.03–32.43)	7.55×10^{-9}	3.77×10^{-8}
Moderna	Dermatological	0	2	291	2817	N/E	N/E	1.000	1.000
Moderna	Cardiac	0	0	291	2817	N/E	N/E	N/E	N/E
AstraZeneca/Covishield	Neurological	5	48	367	2817	0.80 (0.32–2.01)	0.80 (0.32–2.00)	0.828	0.881
AstraZeneca/Covishield	Endocrine	5	2	367	2817	19.44 (3.76–100.57)	19.19 (3.74–98.55)	0.000342	0.000854
AstraZeneca/Covishield	Rheumatological	12	102	367	2817	0.90 (0.49–1.65)	0.90 (0.50–1.63)	0.881	0.881
AstraZeneca/Covishield	Hematological	9	9	367	2817	7.84 (3.09–19.89)	7.68 (3.07–19.21)	6.13×10^{-5}	0.000306
AstraZeneca/Covishield	Dermatological	2	2	367	2817	7.71 (1.08–54.92)	7.68 (1.08–54.33)	0.0679	0.113
AstraZeneca/Covishield	Cardiac	0	0	367	2817	N/E	N/E	N/E	N/E
Janssen	Neurological	0	48	17	2817	N/E	N/E	1.000	1.000
Janssen	Endocrine	0	2	17	2817	N/E	N/E	1.000	1.000
Janssen	Rheumatological	0	102	17	2817	N/E	N/E	1.000	1.000
Janssen	Hematological	0	9	17	2817	N/E	N/E	1.000	1.000
Janssen	Dermatological	0	2	17	2817	N/E	N/E	1.000	1.000

Janssen	Cardiac	0	0	17	2817	N/E	N/E	N/E	N/E
Multiple COVID products	Neurological	5	48	85	2817	3.61 (1.40–9.30)	3.45 (1.41–8.45)	0.0182	0.0456
Multiple COVID products	Endocrine	0	2	85	2817	N/E	N/E	1.000	1.000
Multiple COVID products	Rheumatological	3	102	85	2817	0.97 (0.30–3.13)	0.97 (0.32–3.01)	1.000	1.000
Multiple COVID products	Hematological	4	9	85	2817	15.41 (4.65–51.07)	14.73 (4.63–46.89)	0.000401	0.002
Multiple COVID products	Dermatological	0	2	85	2817	N/E	N/E	1.000	1.000
Multiple COVID products	Cardiac	0	0	85	2817	N/E	N/E	N/E	N/E

Table S7. Age-Interaction Analysis Based on ROR Ratios.

Category	ROR <40	ROR 40–64	ROR ≥65	<40 vs 40–64 ROR Ratio	*p* Interaction	≥65 vs 40–64 ROR Ratio	*p* Interaction	<40 vs ≥65 ROR Ratio	*p* Interaction	*p* Trend
Neurological	3.26	1.36	0.26	2.40 (0.90–6.42)	0.0805	0.19 (0.06–0.57)	0.0029	12.53 (3.63–43.29)	0.000064	0.000087
Endocrine	N/E	1.53	N/E	N/E	N/E	N/E	N/E	N/E	N/E	N/E
Rheumatological	0.47	0.35	0.55	1.36 (0.32–5.73)	0.6773	1.58 (0.77–3.22)	0.2116	0.86 (0.20–3.69)	0.8410	0.3565
Hematological	7.81	1.23	7.84	6.36 (0.60–68.01)	0.1259	6.38 (1.05–38.74)	0.0441	1.00 (0.08–12.63)	0.9984	0.5325
Dermatological	N/E	2.77	7.76	N/E	N/E	2.80 (0.16–50.44)	0.4848	N/E	N/E	N/E
Cardiac	N/E	N/E	N/E	N/E	N/E	N/E	N/E	N/E	N/E	N/E

Table S8. Preferred Term-Level Disproportionality Analysis of Autoimmune Events.

Preferred Term	Category	A: COVID event	C: Comparator event	ROR (95% CI)	PRR (95% CI)	Fisher–BH p	Interpretation
Autoimmune myocarditis	Cardiac	0	0	N/E	N/E	N/A	Not estimable; no hypothesis test
Autoimmune pericarditis	Cardiac	0	0	N/E	N/E	N/A	Not estimable; no hypothesis test

Alopecia areata	Dermatological	7	0	N/E	N/E	0.0787	No adjusted exact imbalance; ROR/PRR not estimable
Autoimmune dermatitis	Dermatological	0	0	N/E	N/E	N/A	Not estimable; no hypothesis test
Bullous pemphigoid	Dermatological	0	0	N/E	N/E	N/A	Not estimable; no hypothesis test
Cutaneous lupus erythematosus	Dermatological	8	0	N/E	N/E	0.0521	No adjusted exact imbalance; ROR/PRR not estimable
Lichen planus	Dermatological	7	0	N/E	N/E	0.0787	No adjusted exact imbalance; ROR/PRR not estimable
Morphea	Dermatological	0	0	N/E	N/E	N/A	Not estimable; no hypothesis test
Pemphigus	Dermatological	8	0	N/E	N/E	0.0521	No adjusted exact imbalance; ROR/PRR not estimable
Vitiligo	Dermatological	1	2	0.34 (0.03–3.71)	0.34 (0.03–3.70)	0.6141	No adjusted positive signal
Addison's disease	Endocrine	0	0	N/E	N/E	N/A	Not estimable; no hypothesis test
Autoimmune adrenalitis	Endocrine	0	0	N/E	N/E	N/A	Not estimable; no hypothesis test
Autoimmune hypophysitis	Endocrine	0	0	N/E	N/E	N/A	Not estimable; no hypothesis test
Autoimmune insulin syndrome	Endocrine	0	0	N/E	N/E	N/A	Not estimable; no hypothesis test
Autoimmune thyroiditis	Endocrine	5	2	1.68 (0.33–8.67)	1.68 (0.33–8.66)	0.7364	No adjusted positive signal
Basedow's disease	Endocrine	7	0	N/E	N/E	0.0787	No adjusted exact imbalance; ROR/PRR not estimable
Graves' disease	Endocrine	2	0	N/E	N/E	0.6094	No adjusted exact imbalance; ROR/PRR not estimable
Hashimoto's thyroiditis	Endocrine	0	0	N/E	N/E	N/A	Not estimable; no hypothesis test

Polyglandular autoimmune syndrome type I	Endocrine	0	0	N/E	N/E	N/A	Not estimable; no hypothesis test
Polyglandular autoimmune syndrome type II	Endocrine	0	0	N/E	N/E	N/A	Not estimable; no hypothesis test
Type 1 diabetes mellitus	Endocrine	2	0	N/E	N/E	0.6094	No adjusted exact imbalance; ROR/PRR not estimable
Acquired haemophilia	Hematological	20	1	13.50 (1.81–100.67)	13.44 (1.81–100.11)	0.0032	Positive finite disproportionality signal
Autoimmune haemolytic anaemia	Hematological	5	0	N/E	N/E	0.138	No adjusted exact imbalance; ROR/PRR not estimable
Autoimmune neutropenia	Hematological	0	0	N/E	N/E	N/A	Not estimable; no hypothesis test
Evans syndrome	Hematological	0	0	N/E	N/E	N/A	Not estimable; no hypothesis test
Immune thrombocytopenia	Hematological	37	8	3.13 (1.45–6.73)	3.11 (1.45–6.67)	0.0088	Positive finite disproportionality signal
Pure red cell aplasia	Hematological	0	0	N/E	N/E	N/A	Not estimable; no hypothesis test
Acute disseminated encephalomyelitis	Neurological	0	8	N/E	N/E	0.0037	Adjusted exact imbalance; lower reporting; ROR/PRR not estimable
Autoimmune encephalitis	Neurological	0	0	N/E	N/E	N/A	Not estimable; no hypothesis test
Autoimmune neuropathy	Neurological	0	0	N/E	N/E	N/A	Not estimable; no hypothesis test
Guillain-Barre syndrome	Neurological	10	24	0.28 (0.13–0.58)	0.28 (0.13–0.58)	0.0032	Adjusted lower-reporting finding
Miller Fisher syndrome	Neurological	9	0	N/E	N/E	0.0369	Adjusted exact imbalance; higher reporting; ROR/PRR not estimable
Multiple sclerosis	Neurological	55	12	3.11 (1.66–5.81)	3.08 (1.65–5.74)	0.0018	Positive finite disproportionality signal
Myasthenia gravis	Neurological	19	2	6.41 (1.49–27.54)	6.39 (1.49–27.39)	0.0117	Positive finite disproportionality signal

Neuromyelitis optica spectrum disorder	Neurological	1	2	0.34 (0.03–3.71)	0.34 (0.03–3.70)	0.6141	No adjusted positive signal
Optic neuritis	Neurological	22	3	4.95 (1.48–16.55)	4.93 (1.48–16.45)	0.0117	Positive finite disproportionality signal
Transverse myelitis	Neurological	0	0	N/E	N/E	N/A	Not estimable; no hypothesis test
Ankylosing spondylitis	Rheumatological	3	5	0.40 (0.10–1.69)	0.40 (0.10–1.69)	0.3791	No adjusted positive signal
Behcet's syndrome	Rheumatological	0	0	N/E	N/E	N/A	Not estimable; no hypothesis test
Dermatomyositis	Rheumatological	0	1	N/E	N/E	0.5168	No adjusted exact imbalance; ROR/PRR not estimable
Juvenile idiopathic arthritis	Rheumatological	1	6	0.11 (0.01–0.93)	0.11 (0.01–0.93)	0.0474	Adjusted lower-reporting finding
Polymyalgia rheumatica	Rheumatological	5	9	0.37 (0.12–1.11)	0.37 (0.13–1.11)	0.1476	No adjusted positive signal
Polymyositis	Rheumatological	1	0	N/E	N/E	1.000	No adjusted exact imbalance; ROR/PRR not estimable
Reactive arthritis	Rheumatological	0	0	N/E	N/E	N/A	Not estimable; no hypothesis test
Rheumatoid arthritis	Rheumatological	38	71	0.35 (0.24–0.53)	0.36 (0.24–0.53)	4.64×10^{-6}	Adjusted lower-reporting finding
Sjogren's syndrome	Rheumatological	2	9	0.15 (0.03–0.69)	0.15 (0.03–0.69)	0.0288	Adjusted lower-reporting finding
Systemic lupus erythematosus	Rheumatological	42	19	1.49 (0.87–2.57)	1.49 (0.87–2.55)	0.2688	No adjusted positive signal
Systemic sclerosis	Rheumatological	0	0	N/E	N/E	N/A	Not estimable; no hypothesis test

Abbreviations: ROR, reporting odds ratio; PRR, proportional reporting ratio; CI, confidence interval; BH, Benjamini–Hochberg; A, number of PT-coded reports listing a COVID-19 vaccine as a suspect product; C, number of comparator PT reports; N/E, not estimable; N/A, not applicable. Raw p values were obtained using two-sided Fisher exact tests. Benjamini–Hochberg adjustment was applied across the 27 non-degenerate strict PT-level tables. Zero-cell results with adjusted Fisher support are reported as exact imbalances rather than finite ROR/PRR signals.

Table S9. Concordance Between Category-Level and PT-Level Signal Detection.

Autoimmune Category	Category-Level ROR	Category-Level PRR	PT-Level Pattern	Interpretation
Hematological	4.68	4.63	Acquired haemophilia and immune thrombocytopenia were positive finite PT-level signals.	Strong concordance between category-level and PT-level signals.
Dermatological	10.49	10.42	Several dermatological PTs were COVID-only or had zero comparator events; finite PT-level RORs were mostly not estimable.	Sparse-cell concordance: category signal supported by multiple COVID-only PTs, but PT-specific finite ROR inference was limited.
Neurological	1.63	1.61	Multiple sclerosis, myasthenia gravis, and optic neuritis were positive finite signals. Miller Fisher syndrome showed an adjusted higher exact imbalance, while Guillain-Barre syndrome and acute disseminated encephalomyelitis showed lower reporting/exact imbalance.	Partial concordance with heterogeneous finite and zero-cell PT-level directions.
Endocrine	5.39	5.38	No adjusted finite PT-level positive signal; several endocrine PTs had zero comparator events.	Sparse-event concordance: positive category-level signal, but PT-level estimates were limited by zero comparator cells.
Rheumatological	0.60	0.61	No strict rheumatological PT had an adjusted positive finite signal; rheumatoid arthritis, Sjogren's syndrome, and juvenile idiopathic arthritis showed adjusted lower reporting.	Concordant lower reporting proportion versus comparator.
Cardiac	N/E	N/E	No strict cardiac PT-level events were observed.	Concordant negative under the strict autoimmune definition.

Table S10. Discordance Between Category-Level and PT-Level Signal Detection.

Autoimmune Category	Category-Level Finding	PT-Level Finding	Type of Discordance	Interpretation
Dermatological	Positive category-level signal (ROR 10.49; PRR 10.42).	Several PTs were COVID-only, making finite uncorrected PT-level ROR/PRR not estimable.	Sparse-cell discordance	The category-level signal is directionally supported, but PT-specific inference is limited by zero comparator cells.
Endocrine	Positive category-level signal (ROR 5.39; PRR 5.38).	No adjusted finite PT-level positive signal; multiple PTs had zero comparator events.	Sparse-event discordance	The category signal is driven by small counts and non-estimable PT-level cells.
Neurological	Positive category-level signal (ROR 1.63; PRR 1.61).	Multiple sclerosis, myasthenia gravis, and optic neuritis were positive finite signals; Miller Fisher syndrome showed a higher adjusted exact imbalance, whereas Guillain-Barre syndrome and acute disseminated encephalomyelitis showed lower reporting.	Within-category heterogeneity	The aggregate neurological signal contains clinically distinct finite and zero-cell directions.
Cardiac	No strict category-level events; ROR/PRR not estimable.	No strict cardiac PT-level signal.	Definition-level contrast	The broad cardiac signal in sensitivity analysis reflects generic cardiac terms and should not be interpreted as a strict autoimmune cardiac signal.

Table S11. Sensitivity Analyses of Autoimmune Safety Signals.

Scenario	Category	A: COVID Event	C: Comparator Event	ROR (95% CI)	PRR (95% CI)	Fisher–BH p
Strict primary PS+SS	Neurological	115	48	1.63 (1.16-2.29)	1.61 (1.15-2.25)	0.00572
Strict primary PS+SS	Endocrine	16	2	5.39 (1.24-23.48)	5.38 (1.24-23.37)	0.0138
Strict primary PS+SS	Rheumatological	92	102	0.60 (0.45-0.80)	0.61 (0.46-0.80)	0.000764

Strict primary PS+SS	Hematological	62	9	4.68 (2.32-9.44)	4.63 (2.31-9.30)	2.73×10^{-6}
Strict primary PS+SS	Dermatological	31	2	10.49 (2.51-43.86)	10.42 (2.50-43.50)	4.42×10^{-5}
Strict primary PS+SS	Cardiac	0	0	N/E	N/E	N/E
Broad PT sensitivity	Neurological	117	67	1.18 (0.87-1.60)	1.17 (0.87-1.58)	0.322
Broad PT sensitivity	Endocrine	16	2	5.39 (1.24-23.48)	5.38 (1.24-23.37)	0.0276
Broad PT sensitivity	Rheumatological	128	116	0.73 (0.57-0.95)	0.74 (0.58-0.95)	0.0298
Broad PT sensitivity	Hematological	148	41	2.48 (1.75-3.51)	2.43 (1.72-3.42)	1.95×10^{-7}
Broad PT sensitivity	Dermatological	63	29	1.47 (0.94-2.28)	1.46 (0.94-2.26)	0.129
Broad PT sensitivity	Cardiac	102	14	4.99 (2.85-8.75)	4.90 (2.81-8.54)	1.39×10^{-10}
Strict peak window	Neurological	28	31	0.53 (0.31-0.88)	0.53 (0.32-0.88)	0.0182
Strict peak window	Endocrine	7	0	N/E	N/E	0.0514
Strict peak window	Rheumatological	26	57	0.26 (0.16-0.42)	0.27 (0.17-0.42)	2.63×10^{-8}
Strict peak window	Hematological	37	3	7.34 (2.26-23.85)	7.25 (2.24-23.47)	6.87×10^{-5}
Strict peak window	Dermatological	23	0	N/E	N/E	6.87×10^{-5}
Strict peak window	Cardiac	0	0	N/E	N/E	N/E
Strict brand-only comparator	Neurological	115	11	3.45 (1.85-6.42)	3.38 (1.83-6.26)	1.95×10^{-5}
Strict brand-only comparator	Endocrine	16	0	N/E	N/E	0.0219
Strict brand-only comparator	Rheumatological	92	39	0.76 (0.52-1.11)	0.76 (0.53-1.10)	0.151
Strict brand-only comparator	Hematological	62	1	20.33 (2.82-146.77)	20.05 (2.78-144.43)	4.09×10^{-6}
Strict brand-only comparator	Dermatological	31	0	N/E	N/E	0.000379
Strict brand-only comparator	Cardiac	0	0	N/E	N/E	N/E

Strict PS-only	Neurological	7	4	1.16 (0.34-3.97)	1.15 (0.34-3.92)	1.000
Strict PS-only	Endocrine	2	0	N/E	N/E	0.651
Strict PS-only	Rheumatological	18	1	12.15 (1.62-91.32)	11.88 (1.59-88.69)	0.00686
Strict PS-only	Hematological	4	0	N/E	N/E	0.39
Strict PS-only	Dermatological	2	0	N/E	N/E	0.651
Strict PS-only	Cardiac	0	0	N/E	N/E	N/E

Abbreviations: A, event count among reports listing a COVID-19 vaccine as a suspect product; C, comparator event count; ROR, reporting odds ratio; PRR, proportional reporting ratio; CI, confidence interval; BH, Benjamini–Hochberg; N/E, not estimable. All displayed adjusted p values are based on two-sided Fisher exact tests. Note. A, COVID-19 vaccine reports with the event; C, comparator reports with the event; PS+SS, primary suspect plus secondary suspect; PS-only, primary suspect only; ROR, reporting odds ratio; PRR, proportional reporting ratio; CI, confidence interval; BH, Benjamini–Hochberg; N/E, not estimable; N/A, not applicable. Primary strict and broad analyses used 4,191 COVID reports and 2,817 comparator reports; peak-window analyses used 2,520 and 1,481; brand-only comparator analyses used 4,191 and 1,355; and PS-only analyses used 735 and 485. Fisher exact p values were adjusted separately within each non-degenerate scenario family. A supported zero-cell result denotes an exact imbalance and does not provide a finite ROR/PRR estimate.

Table S12. Prespecified Product Terms and Rules Used for Vaccine Cohort Identification.

Cohort	Identification Rule	Prespecified Term
COVID-19 vaccine cohort	Exact standardized product term; PS or SS DRUG role required	TOZINAMERAN
COVID-19 vaccine cohort	Exact standardized product term; PS or SS DRUG role required	ELASOMERAN
COVID-19 vaccine cohort	Exact standardized product term; PS or SS DRUG role required	VAXZEVRIA
COVID-19 vaccine cohort	Exact standardized product term; PS or SS DRUG role required	NUVAXOVID
COVID-19 vaccine cohort	Exact standardized product term; PS or SS DRUG role required	COVISHIELD
COVID-19 vaccine cohort	Exact standardized product term; PS or SS DRUG role required	AD26.COV2
COVID-19 vaccine cohort	Exact standardized product term; PS or SS DRUG role required	SPIKEVAX
COVID-19 vaccine cohort	Exact standardized product term; PS or SS DRUG role required	COMIRNATY
COVID-19 vaccine cohort	Exact standardized product term; PS or SS DRUG role required	BNT162B2
COVID-19 vaccine cohort	Exact standardized product term; PS or SS DRUG role required	MRNA-1273
Traditional non-COVID vaccine comparator	Generic substring; PS or SS DRUG role required	VACCINE
Traditional non-COVID vaccine comparator	Generic substring; PS or SS DRUG role required	VAX
Traditional non-COVID vaccine comparator	Generic substring; PS or SS DRUG role required	TOXOID

Traditional non-COVID vaccine comparator	Curated traditional-vaccine brand; PS or SS DRUG role required	FLUZONE
Traditional non-COVID vaccine comparator	Curated traditional-vaccine brand; PS or SS DRUG role required	FLUARIX
Traditional non-COVID vaccine comparator	Curated traditional-vaccine brand; PS or SS DRUG role required	FLUVIRIN
Traditional non-COVID vaccine comparator	Curated traditional-vaccine brand; PS or SS DRUG role required	FLUCELVAX
Traditional non-COVID vaccine comparator	Curated traditional-vaccine brand; PS or SS DRUG role required	AFLURIA
Traditional non-COVID vaccine comparator	Curated traditional-vaccine brand; PS or SS DRUG role required	FLUBLOCK
Traditional non-COVID vaccine comparator	Curated traditional-vaccine brand; PS or SS DRUG role required	FLULAVAL
Traditional non-COVID vaccine comparator	Curated traditional-vaccine brand; PS or SS DRUG role required	FLUMIST
Traditional non-COVID vaccine comparator	Curated traditional-vaccine brand; PS or SS DRUG role required	GARDASIL
Traditional non-COVID vaccine comparator	Curated traditional-vaccine brand; PS or SS DRUG role required	CERVARIX
Traditional non-COVID vaccine comparator	Curated traditional-vaccine brand; PS or SS DRUG role required	PREVNAR
Traditional non-COVID vaccine comparator	Curated traditional-vaccine brand; PS or SS DRUG role required	PNEUMOVAX
Traditional non-COVID vaccine comparator	Curated traditional-vaccine brand; PS or SS DRUG role required	VAXNEUVANCE
Traditional non-COVID vaccine comparator	Curated traditional-vaccine brand; PS or SS DRUG role required	SHINGRIX
Traditional non-COVID vaccine comparator	Curated traditional-vaccine brand; PS or SS DRUG role required	ZOSTAVAX
Traditional non-COVID vaccine comparator	Curated traditional-vaccine brand; PS or SS DRUG role required	VARIVAX
Traditional non-COVID vaccine comparator	Curated traditional-vaccine brand; PS or SS DRUG role required	PROQUAD
Traditional non-COVID vaccine comparator	Curated traditional-vaccine brand; PS or SS DRUG role required	MENACTRA
Traditional non-COVID vaccine comparator	Curated traditional-vaccine brand; PS or SS DRUG role required	MENVEO

Traditional non-COVID vaccine comparator	Curated traditional-vaccine brand; PS or SS DRUG role required	TRUMENBA
Traditional non-COVID vaccine comparator	Curated traditional-vaccine brand; PS or SS DRUG role required	BEXSERO
Traditional non-COVID vaccine comparator	Curated traditional-vaccine brand; PS or SS DRUG role required	ROTATEQ
Traditional non-COVID vaccine comparator	Curated traditional-vaccine brand; PS or SS DRUG role required	ROTARIX
Traditional non-COVID vaccine comparator	Curated traditional-vaccine brand; PS or SS DRUG role required	ADACEL
Traditional non-COVID vaccine comparator	Curated traditional-vaccine brand; PS or SS DRUG role required	BOOSTRIX
Traditional non-COVID vaccine comparator	Curated traditional-vaccine brand; PS or SS DRUG role required	TENIVAC
Traditional non-COVID vaccine comparator	Curated traditional-vaccine brand; PS or SS DRUG role required	INFANRIX
Traditional non-COVID vaccine comparator	Curated traditional-vaccine brand; PS or SS DRUG role required	PEDIARIX
Traditional non-COVID vaccine comparator	Curated traditional-vaccine brand; PS or SS DRUG role required	PENTACEL
Traditional non-COVID vaccine comparator	Curated traditional-vaccine brand; PS or SS DRUG role required	ENGERIX
Traditional non-COVID vaccine comparator	Curated traditional-vaccine brand; PS or SS DRUG role required	HAVRIX
Traditional non-COVID vaccine comparator	Curated traditional-vaccine brand; PS or SS DRUG role required	TWINRIX
Traditional non-COVID vaccine comparator	Curated traditional-vaccine brand; PS or SS DRUG role required	RECOMBIVAX
Traditional non-COVID vaccine comparator	Curated traditional-vaccine brand; PS or SS DRUG role required	HEPLISAV
Traditional non-COVID vaccine comparator	Curated traditional-vaccine brand; PS or SS DRUG role required	IPOL
Traditional non-COVID vaccine comparator	Curated traditional-vaccine brand; PS or SS DRUG role required	IMOVAX
Traditional non-COVID vaccine comparator	Curated traditional-vaccine brand; PS or SS DRUG role required	RABAVERT
Traditional non-COVID vaccine comparator	Curated traditional-vaccine brand; PS or SS DRUG role required	IXIARO

Traditional non-COVID vaccine comparator	Curated traditional-vaccine brand; PS or SS DRUG role required	VAXCHORA
Traditional non-COVID vaccine comparator	Curated traditional-vaccine brand; PS or SS DRUG role required	TYPHIM
Traditional non-COVID vaccine comparator	Curated traditional-vaccine brand; PS or SS DRUG role required	VIVOTIF
Traditional non-COVID vaccine comparator	Curated traditional-vaccine brand; PS or SS DRUG role required	JYNNEOS
Traditional non-COVID vaccine comparator	Curated traditional-vaccine brand; PS or SS DRUG role required	ACAM2000

Note: Any report containing a prespecified COVID-19 vaccine term was assigned to the COVID-19 vaccine cohort and excluded from the traditional-vaccine comparator, including reports with coadministered COVID-19 and non-COVID vaccines. Bare manufacturer tokens were not used. All exposure terms were evaluated in PS/SS DRUG records for the primary analysis.

Table S13. Composition of the protocol-screened traditional non-COVID vaccine comparator cohort by harmonized vaccine class and product.

Panel A. Mutually exclusive vaccine-class composition.

Harmonized comparator class	Reports, n	% of 2,817	Serious, n	Serious, %	Class-Specific Analysis
Influenza	684	24.3	527	77.0	Yes
Pneumococcal	281	10.0	248	88.3	Yes
Meningococcal	318	11.3	298	93.7	Yes
BCG/tuberculosis	139	4.9	136	97.8	Yes
Herpes zoster	289	10.3	218	75.4	Yes
Varicella	92	3.3	15	16.3	No
Varicella/zoster unspecified	30	1.1	9	30.0	No
MMR	93	3.3	80	86.0	No
MMR-varicella combination	65	2.3	12	18.5	No
Tetanus/diphtheria/pertussis-containing	123	4.4	84	68.3	Yes
HPV	45	1.6	41	91.1	No
Hepatitis A/B	83	2.9	74	89.2	No
Polio	12	0.4	0	0.0	No
Haemophilus influenzae type b	10	0.4	3	30.0	No
Rotavirus	4	0.1	1	25.0	No
Rabies	24	0.9	13	54.2	No

Mpox/smallpox	6	0.2	1	16.7	No
Travel/other prophylactic	15	0.5	11	73.3	No
Multiple traditional vaccine classes	298	10.6	245	82.2	No
Non-vaccine false-positive substring	147	5.2	38	25.9	N/A
Unspecified vaccine term	47	1.7	41	87.2	N/A
Therapeutic/experimental vaccine term	10	0.4	9	90.0	N/A
COVID-19 vaccine term leakage	2	0.1	1	50.0	N/A

Note: Rows in Panel A are mutually exclusive and sum to the protocol-screened comparator denominator of 2,817. Reports containing products from more than one traditional-vaccine class are shown as 'Multiple traditional vaccine classes.' The four yellow audit rows total 206 reports and were excluded from the product-classifiable restricted comparator (2,817 - 206 = 2,611). Seriousness denotes at least one serious FAERS OUTC code (death, life-threatening, hospitalization, disability, congenital anomaly, or other serious outcome).

Panel B. Harmonized product contributions

Harmonized Product or Product Group	Reports, n	% of 2817
Influenza: generic/unspecified product	626	22.2
PREVNAR	254	9.0
Meningococcal: generic/unspecified product	218	7.7
SHINGRIX	184	6.5
Tetanus/diphtheria/pertussis-containing: generic/unspecified product	183	6.5
BCG/tuberculosis: generic/unspecified product	155	5.5
FLUMIST	141	5.0
Pneumococcal: generic/unspecified product	137	4.9
VARIVAX	116	4.1
ZOSTAVAX	113	4.0
BEXSERO	109	3.9
PROQUAD	83	2.9
MMR: generic/unspecified product	75	2.7
PNEUMOVAX	66	2.3
Hepatitis A/B: generic/unspecified product	63	2.2
GARDASIL	42	1.5
M-M-RVAXPRO	39	1.4
Varicella/zoster unspecified: generic/unspecified product	38	1.3
VAXIGRIP	37	1.3

ENGERIX-B	33	1.2
FLUZONE	31	1.1
BOOSTRIX	26	0.9
IPOL	21	0.7
ADACEL	20	0.7
Herpes zoster: generic/unspecified product	19	0.7
Rabies: generic/unspecified product	16	0.6
MENACTRA	14	0.5
REVAXIS	14	0.5
Travel/other prophylactic: generic/unspecified product	14	0.5
FLUARIX	12	0.4
HPV: generic/unspecified product	12	0.4
INFANRIX	12	0.4
ROTARIX	12	0.4
FLULAVAL	11	0.4
REPEVAX	11	0.4
PEDVAXHIB	10	0.4
PENTACEL	9	0.3
Polio: generic/unspecified product	9	0.3
FLUCELVAX	8	0.3
TENIVAC	7	0.2
CERVARIX	6	0.2
IMOVAX RABIES	6	0.2
MENVEO	6	0.2

Panel B continued. Harmonized product contributions

**Harmonized Product or Product Group**	**Reports, n**	**% of 2,817**
Mpox/smallpox: generic/unspecified product	5	0.2
TWINRIX	5	0.2
Haemophilus influenzae type b: generic/unspecified product	4	0.1
RABAVERT	4	0.1
Varicella: generic/unspecified product	4	0.1
HBVAXPRO	3	0.1
ROTATEQ	3	0.1

Rotavirus: generic/unspecified product	3	0.1
TYPHIM VI	3	0.1
AVAXIM	2	0.1
MMR-varicella combination: generic/unspecified product	2	0.1
TRUMENBA	2	0.1
YF-VAX	2	0.1
FLUVAX	1	0.0
FLUVIRIN	1	0.0
HAVRIX	1	0.0
IMOVAX POLIO	1	0.0
IXIARO	1	0.0
JYNNEOS	1	0.0
PEDIARIX	1	0.0
RECOMBIVAX HB	1	0.0
TETAVAX	1	0.0
VAXELIS	1	0.0
VAXNEUVANCE	1	0.0
VIVOTIF	1	0.0

Note: Class-specific sensitivity analyses were restricted to mutually exclusive single-class comparator groups containing ≥100 reports. Accordingly, six classes were analyzed separately in Table S14, Panel B: influenza, pneumococcal, meningococcal, BCG/tuberculosis, herpes zoster, and tetanus/diphtheria/pertussis-containing vaccines. "No" indicates an eligible vaccine class with fewer than 100 reports or a multiple-class group; "N/A" indicates audit or exclusion categories that were not eligible for class-specific analysis.

Table S14. Strict autoimmune category-level disproportionality estimates using pooled and major traditional-vaccine comparator definitions.

Panel A. Protocol-defined pooled comparator and product-classifiable restricted sensitivity comparator

Comparator Definition	Comparator N	Category	COVID Event n	Comparator Event n	ROR (95% CI)	PRR (95% CI)	Fisher–BH p	Interpretation
Protocol pooled comparator	2817	Neurological	115	48	1.63 (1.16-2.29)	1.61 (1.15-2.25)	0.00572	Positive signal vs this comparator class
Protocol pooled comparator	2817	Endocrine	16	2	5.39 (1.24-23.48)	5.38 (1.24-23.37)	0.0138	Positive signal; sparse/imprecise

Protocol pooled comparator	2817	Rheumatological	92	102	0.60 (0.45-0.80)	0.61 (0.46-0.80)	0.000764	Lower reporting vs this comparator class
Protocol pooled comparator	2817	Hematological	62	9	4.68 (2.32-9.44)	4.63 (2.31-9.30)	2.73×10^{-6}	Positive signal vs this comparator class
Protocol pooled comparator	2817	Dermatological	31	2	10.49 (2.51-43.86)	10.42 (2.50-43.50)	4.42×10^{-5}	Positive signal; sparse/imprecise
Protocol pooled comparator	2817	Cardiac	0	0	N/E	N/E	N/E	Not estimable
Product-classifiable restricted comparator	2611	Neurological	115	47	1.54 (1.09-2.17)	1.52 (1.09-2.13)	0.0154	Positive signal vs this comparator class
Product-classifiable restricted comparator	2611	Endocrine	16	2	5.00 (1.15-21.76)	4.98 (1.15-21.66)	0.0154	Positive signal; sparse/imprecise
Product-classifiable restricted comparator	2611	Rheumatological	92	98	0.58 (0.43-0.77)	0.58 (0.44-0.77)	0.000327	Lower reporting vs this comparator class
Product-classifiable restricted comparator	2611	Hematological	62	9	4.34 (2.15-8.75)	4.29 (2.14-8.62)	9.71×10^{-6}	Positive signal vs this comparator class
Product-classifiable restricted comparator	2611	Dermatological	31	2	9.72 (2.32-40.65)	9.66 (2.31-40.32)	9.67×10^{-5}	Positive signal; sparse/imprecise
Product-classifiable restricted comparator	2611	Cardiac	0	0	N/E	N/E	N/E	Not estimable

Note: COVID cohort denominator = 4191. The primary protocol-defined traditional-vaccine comparator denominator = 2817. The restricted sensitivity comparator retained the 2,611 reports classifiable to a traditional-vaccine product or class after exact-token review. ROR, reporting odds ratio; PRR, proportional reporting ratio; CI, confidence interval; BH, Benjamini-Hochberg; N/E, not estimable from the uncorrected 2 x 2 table. Adjusted p values were calculated within each comparator definition across the six autoimmune categories.

Panel B. Analyses using the six largest single vaccine classes

Comparator Class	Class N	Category	COVID Event n	Class Event n	ROR (95% CI)	PRR (95% CI)	Fisher–BH p	Interpretation
Influenza	684	Neurological	115	27	0.69 (0.45-1.05)	0.70 (0.46-1.05)	0.144	No adjusted signal; sparse/exploratory where applicable
Influenza	684	Endocrine	16	1	2.62 (0.35-19.77)	2.61 (0.35-19.66)	0.496	No adjusted signal; sparse/exploratory where applicable
Influenza	684	Rheumatological	92	34	0.43 (0.29-0.64)	0.44 (0.30-0.65)	0.0006	Adjusted lower reporting vs this comparator class
Influenza	684	Hematological	62	6	1.70 (0.73-3.94)	1.69 (0.73-3.88)	0.362	No adjusted signal; sparse/exploratory where applicable
Influenza	684	Dermatological	31	1	5.09 (0.69-37.35)	5.06 (0.69-37.00)	0.144	No adjusted signal; sparse/exploratory where applicable
Influenza	684	Cardiac	0	0	N/E	N/E	N/E	Not estimable
Pneumococcal	281	Neurological	115	1	7.90 (1.10-56.77)	7.71 (1.08-55.00)	0.0496	Adjusted positive signal; sparse/imprecise
Pneumococcal	281	Endocrine	16	1	1.07 (0.14-8.12)	1.07 (0.14-8.06)	1.000	No adjusted signal; sparse/exploratory where applicable
Pneumococcal	281	Rheumatological	92	7	0.88 (0.40-1.91)	0.88 (0.41-1.88)	0.844	No adjusted signal; sparse/exploratory where applicable
Pneumococcal	281	Hematological	62	1	4.20 (0.58-30.43)	4.16 (0.58-29.87)	0.433	No adjusted signal; sparse/exploratory where applicable
Pneumococcal	281	Dermatological	31	0	N/E	N/E	0.433	No adjusted exact imbalance; ROR/PRR N/E
Pneumococcal	281	Cardiac	0	0	N/E	N/E	N/E	Not estimable
Meningococcal	318	Neurological	115	1	8.94 (1.25-64.25)	8.73 (1.22-62.27)	0.0074	Adjusted positive signal; sparse/imprecise
Meningococcal	318	Endocrine	16	0	N/E	N/E	0.623	No adjusted exact imbalance; ROR/PRR N/E

Meningococcal	318	Rheumatological	92	0	N/E	N/E	0.0074	Adjusted exact imbalance; higher reporting; ROR/PRR N/E
Meningococcal	318	Hematological	62	0	N/E	N/E	0.0349	Adjusted exact imbalance; higher reporting; ROR/PRR N/E
Meningococcal	318	Dermatological	31	0	N/E	N/E	0.208	No adjusted exact imbalance; ROR/PRR N/E
Meningococcal	318	Cardiac	0	0	N/E	N/E	N/E	Not estimable
BCG/tuberculosis	139	Neurological	115	0	N/E	N/E	0.264	No adjusted exact imbalance; ROR/PRR N/E
BCG/tuberculosis	139	Endocrine	16	0	N/E	N/E	1.000	No adjusted exact imbalance; ROR/PRR N/E
BCG/tuberculosis	139	Rheumatological	92	0	N/E	N/E	0.305	No adjusted exact imbalance; ROR/PRR N/E
BCG/tuberculosis	139	Hematological	62	0	N/E	N/E	0.445	No adjusted exact imbalance; ROR/PRR N/E
BCG/tuberculosis	139	Dermatological	31	1	1.03 (0.14-7.59)	1.03 (0.14-7.48)	1.000	No adjusted signal; sparse/exploratory where applicable
BCG/tuberculosis	139	Cardiac	0	0	N/E	N/E	N/E	Not estimable
Herpes zoster	289	Neurological	115	1	8.13 (1.13-58.39)	7.93 (1.11-56.57)	0.0162	Adjusted positive signal; sparse/imprecise
Herpes zoster	289	Endocrine	16	0	N/E	N/E	0.62	No adjusted exact imbalance; ROR/PRR N/E
Herpes zoster	289	Rheumatological	92	30	0.19 (0.13-0.30)	0.21 (0.14-0.31)	3.14×10^{-10}	Adjusted lower reporting vs this comparator class
Herpes zoster	289	Hematological	62	0	N/E	N/E	0.0539	No adjusted exact imbalance; ROR/PRR N/E
Herpes zoster	289	Dermatological	31	0	N/E	N/E	0.327	No adjusted exact imbalance; ROR/PRR N/E
Herpes zoster	289	Cardiac	0	0	N/E	N/E	N/E	Not estimable
Tetanus/diphtheria/pertussis-containing	123	Neurological	115	5	0.67 (0.27-1.66)	0.68 (0.28-1.62)	0.706	No adjusted signal; sparse/exploratory where applicable
Tetanus/diphtheria/pertussis-containing	123	Endocrine	16	0	N/E	N/E	1.000	No adjusted exact imbalance; ROR/PRR N/E
Tetanus/diphtheria/pertussis-containing	123	Rheumatological	92	7	0.37 (0.17-0.82)	0.39 (0.18-0.81)	0.11	No adjusted signal; sparse/exploratory where applicable

Tetanus/diphtheria/ pertussis-containing	123	Hematological	62	0	N/E	N/E	0.706	No adjusted exact imbalance; ROR/PRR N/E
Tetanus/diphtheria/ pertussis-containing	123	Dermatological	31	0	N/E	N/E	1.000	No adjusted exact imbalance; ROR/PRR N/E
Tetanus/diphtheria/ pertussis-containing	123	Cardiac	0	0	N/E	N/E	N/E	Not estimable

Note: Class-specific analyses were restricted to mutually exclusive classes with at least 100 reports: influenza, pneumococcal, meningococcal, BCG/tuberculosis, herpes zoster, and tetanus/diphtheria/pertussis-containing vaccines. Each class was compared separately with the same COVID-19 vaccine cohort (n = 4,191). Adjusted p values were calculated within each comparator class across the six autoimmune categories. Estimates based on fewer than five comparator events or containing a zero cell are sparse and should be interpreted descriptively. Continuity-corrected values are retained in the accompanying analytic workbook but are not substituted for uncorrected primary estimates in this table.

Table S15. Serious-Report-Only Sensitivity Analysis of Category-Level Autoimmune Disproportionality. The primary analysis is shown alongside the serious-report-only restriction to make the influence of baseline seriousness asymmetry directly auditable.

Scenario	Category	A: COVID Event	C: Comparator Event	N COVID	N Comparator	ROR (95% CI)	PRR (95% CI)	Fisher–BH p	Interpretation
Primary strict PS+SS	Neurological	115	48	4191	2817	1.63 (1.16-2.29)	1.61 (1.15-2.25)	0.00572	Positive signal; intermediate
Primary strict PS+SS	Endocrine	16	2	4191	2817	5.39 (1.24-23.48)	5.38 (1.24-23.37)	0.0138	Positive signal; sparse/imprecise
Primary strict PS+SS	Rheumatological	92	102	4191	2817	0.60 (0.45-0.80)	0.61 (0.46-0.80)	0.000764	Lower reporting; more stable
Primary strict PS+SS	Hematological	62	9	4191	2817	4.68 (2.32-9.44)	4.63 (2.31-9.30)	2.73×10^{-6}	Positive signal; more stable
Primary strict PS+SS	Dermatological	31	2	4191	2817	10.49 (2.51-43.86)	10.42 (2.50-43.50)	4.42×10^{-5}	Positive signal; sparse/imprecise
Primary strict PS+SS	Cardiac	0	0	4191	2817	N/E	N/E	N/E	Not estimable
Serious reports only	Neurological	115	45	3757	2105	1.45 (1.02-2.05)	1.43 (1.02-2.01)	0.0370	Positive signal; intermediate
Serious reports only	Endocrine	16	2	3757	2105	4.50 (1.03-19.58)	4.48 (1.03-19.48)	0.0341	Positive signal; sparse/imprecise
Serious reports only	Rheumatological	91	100	3757	2105	0.50 (0.37-0.66)	0.51 (0.39-0.67)	1.23×10^{-5}	Lower reporting; more stable

Serious reports only	Hematological	62	9	3757	2105	3.91 (1.94-7.88)	3.86 (1.92-7.75)	3.89×10^{-5}	Positive signal; more stable
Serious reports only	Dermatological	31	2	3757	2105	8.75 (2.09-36.59)	8.68 (2.08-36.25)	0.000256	Positive signal; sparse/imprecise
Serious reports only	Cardiac	0	0	3757	2105	N/E	N/E	N/E	Not estimable

Note: Seriousness was defined as at least one FAERS OUTC code for death, life-threatening outcome, hospitalization, disability, congenital anomaly, or other serious outcome. Serious-only denominators were 3757 COVID-19 vaccine reports and 2105 comparator reports. ROR, reporting odds ratio; PRR, proportional reporting ratio; CI, confidence interval; BH, Benjamini–Hochberg; N/E, not estimable. Raw p values were obtained using two-sided Fisher exact tests and adjusted across the five non-degenerate category tables within each scenario. Robustness tier is descriptive and separate from statistical signal status.

Table S16. Evidence Robustness Classification for Category-Level and Top-Ranked PT Findings.

Level	Finding	A	C	CI fold-Width	Sensitivity Consistency	Robustness tier	Interpretive Basis
Category	Neurological	115	48	1.97	Positive in 2/5 sensitivities	Intermediate	Adequate counts and precision; limited signal consistency
Category	Endocrine	16	2	18.95	Adjusted elevation in 3/5; N/E in 3/5	Sparse/imprecise	Only two comparator events and wide CI
Category	Rheumatological	92	102	1.77	Lower in 3/5; one sparse reversal	More stable	Lower in 3/5 sensitivity analyses; reversed in the sparse PS-only analysis (C=1)
Category	Hematological	62	9	4.06	Positive in 4/5; N/E in 1/5	More stable	Adequate counts/precision and consistent positive direction
Category	Dermatological	31	2	17.48	Adjusted elevation in 3/5; N/E in 3/5	Sparse/imprecise	Only two comparator events and wide CI
Category	Cardiac	0	0	N/E	Strict events absent	Not estimable	No strict cardiac events in either cohort
PT	Acquired haemophilia	20	1	55.62	Parent category positive in 4/5	Sparse/imprecise	High ROR driven by one comparator event and very wide CI
PT	Myasthenia gravis	19	2	18.48	Parent category positive in 2/5	Sparse/imprecise	Two comparator events and wide CI
PT	Optic neuritis	22	3	11.18	Parent category positive in 2/5	Sparse/imprecise	Three comparator events and >10-fold CI width
PT	Immune thrombocytopenia	37	8	4.64	Parent category positive in 4/5	More stable	Adequate counts/precision and consistent parent category

PT	Multiple sclerosis	55	12	3.50	Parent category positive in 2/5	Intermediate	Adequate counts/precision; limited parent-category consistency
PT	Autoimmune thyroiditis	5	2	26.27	Parent category adjusted in 3/5	Sparse/imprecise	Two comparator events and wide CI; no adjusted signal
PT	Systemic lupus erythematosus	42	19	2.95	Parent category lower in 3/5	Intermediate	Adequate precision but PT direction differs from parent category
PT	Ankylosing spondylitis	3	5	16.90	Parent category lower in 3/5	Sparse/imprecise	Only three COVID events and wide CI
PT	Polymyalgia rheumatica	5	9	9.25	Parent category lower in 3/5	More stable	Adequate support/precision; no adjusted positive signal
PT	Rheumatoid arthritis	38	71	2.21	Parent category lower in 3/5	More stable	Well-supported lower-reporting estimate

Note: A and C are COVID-19 and comparator event counts. CI fold-width is the upper divided by the lower 95% confidence limit. Sparse/imprecise indicates A or C <5 or a CI fold-width >10. More stable indicates A and C ≥5, a fold-width ≤10, and retention of the same adjusted category-level direction in at least three of five sensitivity analyses. Other finite estimates are intermediate. For PT rows, consistency refers to the parent category and does not constitute PT-specific replication. Robustness tier does not replace statistical signal status and does not imply causality. The rheumatological PS-only reversal is shown because it indicates suspect-role sensitivity.

Table S17. Formal Interaction Tests for Vaccine Platform, Sex, and Age. Interaction estimates compare subgroup-specific reporting odds ratios directly. A significant result within one subgroup and a non-significant result within another were not considered evidence of interaction.

Panel A. Platform interaction: mRNA versus viral vector

Category	ROR Group 1	ROR Group 2	ROR Ratio	95% CI	Interaction p	BH p	Interpretation
Neurological	1.72	0.76	2.26	0.91-5.56	0.0773	0.193	No adjusted interaction evidence
Endocrine	4.08	18.57	0.22	0.08-0.64	0.00517	0.0258	Adjusted interaction; sparse viral-vector events
Rheumaological	0.57	0.86	0.67	0.36-1.23	0.195	0.244	No adjusted interaction evidence
Hematological	4.40	7.49	0.59	0.29-1.20	0.146	0.243	No adjusted interaction evidence
Dermatological	10.80	7.37	1.47	0.35-6.17	0.602	0.602	No adjusted interaction evidence
Cardiac	N/E	N/E	N/E	N/E	N/E	N/E	Zero event cells

Panel B. Sex interaction: female versus male

Category	ROR Group 1	ROR Group 2	ROR ratio	95% CI	Interaction p	BH p	Interpretation
Neurological	1.76	1.13	1.57	0.74-3.33	0.245	0.491	No adjusted interaction evidence
Endocrine	N/E	N/E	N/E	N/E	N/E	N/E	Zero event cell
Rheumatological	0.44	1.43	0.30	0.14-0.64	0.00176	0.00703	Adjusted interaction; exploratory
Hematological	5.66	4.55	1.24	0.26-6.08	0.787	0.977	No adjusted interaction evidence
Dermatological	8.16	7.82	1.04	0.06-18.49	0.977	0.977	No adjusted interaction evidence
Cardiac	N/E	N/E	N/E	N/E	N/E	N/E	Zero event cells

Panel C. Key age interactions discussed in the main text

Category	Comparison	ROR Ratio	95% CI	Interaction p	BH p	Trend p (BH p)	Interpretation
Neurological	<40 vs 40-64	2.40	0.90-6.42	0.0805	0.201	8.74x10^-5 (0.000262)	Pairwise comparison not supported
Neurological	>=65 vs 40-64	0.19	0.06-0.57	0.00292	0.0146	8.74x10^-5 (0.000262)	Adjusted interaction evidence
Neurological	<40 vs >=65	12.53	3.63-43.29	6.39x10^-5	0.000639	8.74x10^-5 (0.000262)	Adjusted interaction evidence
Hematological	>=65 vs 40-64	6.38	1.05-38.74	0.0441	0.147	0.533 (0.533)	Nominal only; wide CI

Note: ROR ratio is group 1 divided by group 2. Platform groups used a common traditional-vaccine comparator, which cancels algebraically in the ratio. Sex and age ratios used two-sided Wald tests for differences in log RORs. Age trend p values came from inverse-variance weighted regression across ordered age scores. BH adjustment families comprised five platform interactions, four sex interactions, 10 age-pair comparisons, and three age-trend tests. N/E, not estimable. All interaction analyses were exploratory.

Table S18. Independence, Reporting Concentration, and Potential Clinical-Episode Clustering Audit for Acquired Haemophilia.

This post hoc audit examines whether the protocol report-level finding (20 reports listing a COVID-19 vaccine as a suspect product versus 1 traditional-vaccine comparator report) represents broadly independent reports or concentrated parallel submissions.

Panel A. Report-independence and concentration audit

Audit Item	Result	Audit Item	Result
Protocol COVID acquired-haemophilia reports	20	Potential COVID clinical-episode clusters	4
Unique retained COVID CASEIDs	20	COVID reports from France	19/20 (95.0%)
Unique retained COVID PRIMARYIDs	20	COVID reports in 2022Q2	13/20 (65.0%)

COVID CASEIDs with >1 PRIMARYID in downloaded quarters	2	COVID literature-linked reports	0/20
Retained reports confirmed as latest available version	20/20	COVID expedited reports	20/20
Unique retained COVID MFR_NUM values	20	Distinct manufacturer senders	14
Repeated nonblank AUTH_NUM groups	3	Physician reports	14/20 (70.0%)
COVID reports in repeated AUTH_NUM groups	12	Likely cross-cohort parallel comparator report	1/1

Panel 19. clinical-episode clusters.

Cluster	COVID reports	Unique CASEIDs	Event date	Country	COVID Vaccine (lot)	Age / sex	Shared AUTH_NUM (reports)	Senders	Interpretation
AH-C1	9	9	20220330	FR	SPIKEVAX (006G21A)	61.0 / M	FR-AFSSAPS-BS20220924 (6)	9	Probable parallel multi-sender reports of one clinical episode
AH-C2	6	6	20220327	FR	COMIRNATY (FM4574)	96.0 / F	FR-AFSSAPS-AN20221440 (4)	6	Probable parallel multi-sender reports of one clinical episode
AH-C3	4	4	20211028	FR	COMIRNATY (Unknown/not recorded)	67.0 / M	FR-AFSSAPS-LL20220156 (2)	4	Probable parallel multi-sender reports of one clinical episode
AH-C4	1	1	20210101	BE	VAXZEVRIA (Unknown/not recorded)	74.0 / M	Not recorded (0)	1	No within-PT cluster detected

Panel C. Cluster-collapsed disproportionality sensitivity

Scenario	A	C	N COVID	N Comparator	ROR (95% CI)	PRR (95% CI)	Raw Fisher Exact p	Interpretation
Protocol report-level primary analysis	20	1	4191	2817	13.50 (1.81-100.67)	13.44 (1.81-100.11)	0.000468	Original adjusted-positive report-level signal; sparse comparator count.
Collapse repeated nonblank AUTH_NUM within COVID cohort	11	1	4182	2817	7.43 (0.96-57.56)	7.41 (0.96-57.36)	0.0346	Partial duplicate control; blank AUTH_NUM records remain separate.

Collapse four potential COVID clinical-episode clusters; retain comparator as separate	4	1	4175	2817	2.70 (0.30-24.18)	2.70 (0.30-24.14)	0.654	Conservative cluster-collapsed sensitivity; no statistical support.
Four COVID clusters; exclude probable cross-cohort comparator duplicate	4	0	4175	2816	6.08 (0.33-112.91)*	6.07 (0.33-112.72)*	0.153	Comparator event becomes zero; *0.5-corrected estimate is descriptive and highly imprecise.

Panel D. Probable cross-cohort parallel report

The sole comparator report (CASEID 21034850) matched COVID cluster AH-C2 on event date (27 March 2022) and country (France) and shared the co-reported vaccine products REPEVAX and VAXIGRIPTETRA (influenza lot V3H884V), as well as all seven non-vaccine medications in the report profile. These concordant features strongly suggest that the comparator report and the reports in cluster AH-C2 may represent parallel submissions of the same clinical episode; however, this linkage cannot be confirmed without access to the source narratives. Accordingly, the conservative four-versus-one sensitivity analysis retained the comparator event as independent, whereas the alternative four-versus-zero analysis excluded it as a probable cross-cohort duplicate.

Note. Standard FAERS deduplication collapses follow-up versions sharing a CASEID; it cannot guarantee that parallel submissions by different manufacturers receive the same CASEID. Potential episode clusters were defined post hoc from identical event date, country, COVID-vaccine product, and lot (with unknown lot treated as missing), supported by repeated AUTH_NUM values, demographics, and concordant co-reported medication profiles. MFR_NUM and manufacturer sender identify the submitting report, not necessarily the vaccine manufacturer. ROR, reporting odds ratio; PRR, proportional reporting ratio; CI, confidence interval. P values in Panel C are unadjusted and exploratory. The final scenario uses a Haldane-Anscombe 0.5 correction because C=0.

Table S19. Completeness and Descriptive Time to Onset Among Strict-Autoimmune Reports Listing a COVID-19 Vaccine as a Suspect Product.

Panel A. Temporal-field completeness at the unique-report level

Completeness Metric	N	% of 311	Definition
Strict-autoimmune COVID-vaccine reports	311	100.0%	Unique deduplicated report denominator
Exact EVENT_DT (YYYYMMDD)	231	74.3%	Partial or invalid EVENT_DT not imputed
At least one THER row matched to a qualifying COVID-vaccine drug_seq	222	71.4%	Matched by PRIMARYID and DRUG/THER sequence

At least one exact matched vaccine START_DT (YYYYMMDD)	209	67.2%	Partial or invalid START_DT not imputed
Both exact EVENT_DT and exact matched vaccine START_DT	202	65.0%	Eligible for chronological assessment
Chronologically valid interval (>=0 days)	134	43.1%	Most recent qualifying vaccination on/before EVENT_DT
Included in descriptive TTO window (0-365 days)	132	42.4%	Primary descriptive temporal subset
Chronologically valid but >365 days	2	0.6%	Reported separately; excluded from 0-365-day window
All exact matched vaccine START_DT values after EVENT_DT	68	21.9%	Chronologically invalid; excluded

Panel B. Descriptive time to onset within the 0-365-day analysis window.

**Analysis Set / Category**	**Cohort N**	**Valid N**	**Complete**	**Median, d**	**Q1, d**	**Q3, d**	**Range, d**
Unique reports	311	132	42.4%	30.0	10.5	74.0	0-300
Report-category observations	316	134	42.4%	30.0	9.5	73.5	0-300
Neurological	115	44	38.3%	13.0	9.0	46.75	0-300
Endocrine	16	4	25.0%	5.5	0.0	44.25	0-144
Rheumatological	92	15	16.3%	6.0	3.5	23.0	0-128
Hematological	62	51	82.3%	26.0	14.0	90.0	0-160
Dermatological	31	20	64.5%	46.0	40.0	60.75	2-105
Cardiac	0	0	N/E	N/E	N/E	N/E	N/E

Note: Only valid YYYYMMDD dates were analyzed; partial dates were not imputed. For reports with more than one qualifying exact vaccine date, the most recent START_DT on or before EVENT_DT was selected. Two chronologically valid intervals longer than 365 days were excluded from the displayed descriptive window. Category rows are report-category observations and sum to 134 valid observations; 132 unique reports contributed because two reports belonged to two strict autoimmune categories. TTO, time to onset; N/E, not estimable. These summaries are exploratory because temporal completeness varied substantially by category and FAERS dates were not clinically adjudicated.

Table S20. Comparison of Fisher Exact and Yates-Corrected Inference for the Primary Category-Level and Strict PT-Level Families.

Fisher exact testing was used as the primary sparse-table procedure. The Yates-corrected Pearson chi-square analysis is retained here as a labelled sensitivity analysis. Benjamini–Hochberg adjustment was repeated within the same five-test category family and 27-test non-degenerate strict PT family.

Panel A. Primary category-level family

Category	A	C	Fisher Raw p	Fisher–BH p	Yates Raw p	Yates–BH p	Fisher Classification
Neurological	115	48	0.00458	0.00572	0.00594	0.00742	Positive finite signal
Endocrine	16	2	0.0138	0.0138	0.0226	0.0226	Positive finite signal; sparse/imprecise
Rheumatological	92	102	0.000458	0.000764	0.000479	0.000798	Adjusted lower reporting
Hematological	62	9	5.46×10^{-7}	2.73×10^{-6}	3.62×10^{-6}	1.81×10^{-5}	Positive finite signal
Dermatological	31	2	1.77×10^{-5}	4.42×10^{-5}	0.000128	0.000319	Positive finite signal; sparse/imprecise

Panel B. Strict Preferred Term-level family (27 non-degenerate tables).

Preferred Term	A	C	Fisher Raw p	Fisher–BH p	Yates Raw p	Yates–BH p	Fisher Classification
Alopecia areata	7	0	0.0466	0.0787	0.0743	0.125	No adjusted exact imbalance; ROR/PRR not estimable
Cutaneous lupus erythematosus	8	0	0.0251	0.0521	0.0501	0.104	No adjusted exact imbalance; ROR/PRR not estimable
Lichen planus	7	0	0.0466	0.0787	0.0743	0.125	No adjusted exact imbalance; ROR/PRR not estimable
Pemphigus	8	0	0.0251	0.0521	0.0501	0.104	No adjusted exact imbalance; ROR/PRR not estimable
Vitiligo	1	2	0.569	0.614	0.729	0.82	No adjusted positive signal

Autoimmune thyroiditis	5	2	0.709	0.736	0.809	0.873	No adjusted positive signal
Basedow's disease	7	0	0.0466	0.0787	0.0743	0.125	No adjusted exact imbalance; ROR/PRR not estimable
Graves' disease	2	0	0.519	0.609	0.661	0.811	No adjusted exact imbalance; ROR/PRR not estimable
Type 1 diabetes mellitus	2	0	0.519	0.609	0.661	0.811	No adjusted exact imbalance; ROR/PRR not estimable
Acquired haemophilia	20	1	0.000468	0.00316	0.00197	0.0108	Positive finite disproportionality signal
Autoimmune haemolytic anaemia	5	0	0.0869	0.138	0.168	0.252	No adjusted exact imbalance; ROR/PRR not estimable
Immune thrombocytopenia	37	8	0.00195	0.00879	0.00345	0.0155	Positive finite disproportionality signal
Acute disseminated encephalomyelitis	0	8	0.000678	0.00366	0.00199	0.0108	Adjusted exact imbalance; lower reporting; ROR/PRR not estimable
Guillain-Barre syndrome	10	24	0.000369	0.00316	0.000565	0.00509	Adjusted lower-reporting finding
Miller Fisher syndrome	9	0	0.0137	0.0369	0.0339	0.0916	Adjusted exact imbalance; higher reporting; ROR/PRR not estimable
Multiple sclerosis	55	12	0.000134	0.00181	0.000302	0.00408	Positive finite disproportionality signal
Myasthenia gravis	19	2	0.0031	0.0117	0.00809	0.0273	Positive finite disproportionality signal
Neuromyelitis optica spectrum disorder	1	2	0.569	0.614	0.729	0.82	No adjusted positive signal
Optic neuritis	22	3	0.00347	0.0117	0.00744	0.0273	Positive finite disproportionality signal
Ankylosing spondylitis	3	5	0.281	0.379	0.354	0.478	No adjusted positive signal
Dermatomyositis	0	1	0.402	0.517	0.842	0.874	No adjusted exact imbalance; ROR/PRR not estimable
Juvenile idiopathic arthritis	1	6	0.0193	0.0474	0.0383	0.094	Adjusted lower-reporting finding
Polymyalgia rheumatica	5	9	0.0984	0.148	0.117	0.186	No adjusted positive signal
Polymyositis	1	0	1.000	1.000	1.000	1.000	No adjusted exact imbalance; ROR/PRR not estimable

Rheumatoid arthritis	38	71	1.72×10^{-7}	4.64×10^{-6}	1.49×10^{-7}	4.01×10^{-6}	Adjusted lower-reporting finding
Sjogren's syndrome	2	9	0.0096	0.0288	0.0121	0.0362	Adjusted lower-reporting finding
Systemic lupus erythematosus	42	19	0.189	0.269	0.188	0.267	No adjusted positive signal

Note. Fisher and Yates p values are two-sided. Rows with A = C = 0 were excluded from both families. ROR and PRR remain non-estimable when one event cell is zero; in those rows Fisher provides an exact test of the 2 × 2 table rather than a finite effect estimate. The exact reanalysis retained the same five finite adjusted-positive PT findings and additionally supported Miller Fisher syndrome as a higher zero-cell exact imbalance and juvenile idiopathic arthritis as a lower finite finding.

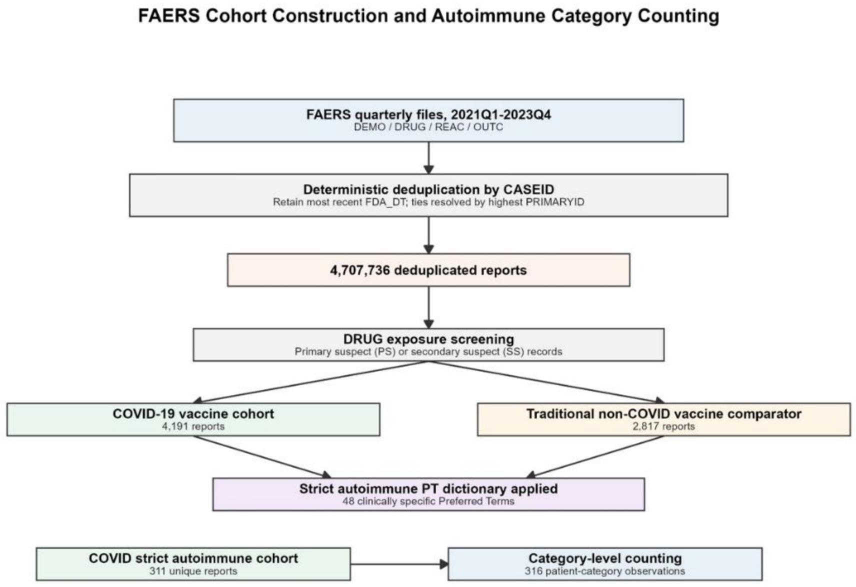

Figure S1. FAERS cohort construction and autoimmune category counting. Deterministic deduplication retained the most recent FDA_DT within each CASEID, with ties resolved by the highest PRIMARYID. Reports containing both COVID-19 and traditional vaccine terms were assigned to the COVID-19 cohort and excluded from the comparator. Multiple PTs within the same autoimmune category were counted once per report, whereas cross-category contributions were retained.
